# Metal–Ligand Cooperation in N–H Activation: Bridging Electron-Pushing Formalism and Energy Descriptors

**DOI:** 10.1021/acs.inorgchem.5c03268

**Published:** 2025-10-22

**Authors:** Daniel Barrena-Espés, Victor Polo, Jorge Echeverría, Ángel Martín Pendás, Julen Munárriz

**Affiliations:** † Departamento de Química Física y Analítica, 16763 Universidad de Oviedo, Oviedo 33006, Spain; ‡ Departamento de Química Física and Instituto de Biocomputación y Física de Sistemas Complejos (BIFI), 16765 Universidad de Zaragoza, Zaragoza 50009, Spain; § Departamento de Química Inorgánica and Instituto de Síntesis Química y Catálisis Homogénea (ISQCH), Universidad de Zaragoza, Pedro Cerbuna 12, Zaragoza 50009, Spain

## Abstract

The activation of N–H bonds is a fundamental step in the synthesis of industrially relevant compounds but remains a challenging process. A promising strategy to address it, introduced by Milstein and co-workers, relies on metal–ligand cooperation, in which N–H activation is coupled with an aromatization–dearomatization process of a *pincer* ligand. In this work, we employ state-of-the-art theoretical methods grounded in quantum chemical topology (QCT) to gain deeper insights into this process. Using the archetypal PNP–Ru­(II) complex reported by Milstein (JACS 2010, 132, 8542), we analyze the electron density rearrangements during N–H activation through the electron localization function and bonding evolution theory. Interacting quantum atoms energy decomposition is further applied to quantify interactions between key groups. The study covers substrates from ammonia to primary amines, revealing that hydrogen transfer occurs as a quasi-protonic species, yielding a Ru–amido complex. The mechanism remains consistent across substrates, with electron-withdrawing groups facilitating the process by stabilizing the NH–R interaction. Additionally, modifying the ligand scaffold with electron-donating substituents enhances charge accumulation at the reactive carbon, improving both kinetics and thermodynamics. Overall, our findings highlight QCT as a powerful framework for guiding the rational design of improved systems.

## Graphical abstract

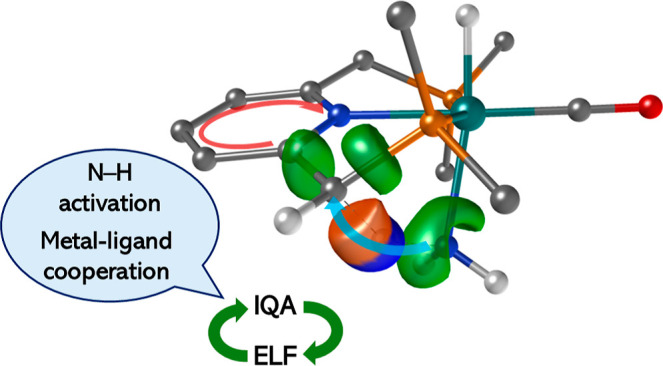

## Introduction

The functionalization of N–H bonds serves as a pivotal step in the synthesis of numerous compounds of significant economic importance, including, for example, fertilizers^1^ and pharmaceuticals.^2^ This transformation has garnered significant attention in chemical research, especially considering ammonia’s status as one of the most widely produced chemicals worldwide.^3^ In this context, substantial efforts have been dedicated to activate N–H bonds,^4,5^ with a particular emphasis on catalytic processes based on transition metal complexes.^6–19^ However, accomplishing this task is far from straightforward due to the tendency of amines to coordinate to the metal center using the nitrogen lone pair instead of the N–H bond that is to be activated, resulting in the formation of stable Werner complexes. Furthermore, the N–H bond exhibits a high strength (about 100 kcal·mol^–1^), and the restricted acidity and moderate basicity of amines discourage the transfer of protons, either into or out of the amine.^6,20,21^ As a consequence, it is unsurprising that the number of processes reported in the literature for activating amines using late metallic precursors be scarce.^22–26^

N–H bond activation has typically been achieved through two main approaches: oxidative addition and metal–ligand cooperation, which are briefly explained below, without prejudice to other potential methods.^27–34^ Oxidative addition of the N–H bond at the metal center proceeds through an inner-sphere mechanism that leads to the homolytic cleavage of the bond and a subsequent increase of the metal center’s oxidation state.^20,35–41^ These processes have also been subjected to scrutiny through theoretical calculations, primarily aimed at comprehending the reaction mechanism and the electronic characteristics of the ligands that govern the kinetics and thermodynamics of the process.^42–45^ A second relevant approach relies on metal–ligand cooperation (MLC) with meticulously designed ligands, which employs a heterolytic mechanism that does not alter the oxidation state of the metal.^14,46^ In this manner, the N–H bond is activated such that the hydrogen atom becomes bonded to the ligand, which is generally based on a pincer scaffold; simultaneously, the “amido” fragment bonds to the metal center. Scheme 1 shows this process for the original PNP-Ru­(II) complex with a dearomatized pyridine-based rest reported by Milstein and co-workers.^47^ As can be deduced from the scheme, the pyridine moiety in the pincer scaffold undergoes rearomatization, which is considered the driving force of the process. The success of the previous case has spurred the development of structural modifications in the dearomatized ligand architecture, including the substitution of CH_2_ arms with NH ones,^12,13^ as well as the utilization of PNP-pincer type phosphaalkene ligands,^48^ among other approaches within the context of MLC.^49–52^ Nonetheless, in contrast to other processes like C–H and O–H activation, progress in the field of N–H activation through MLC remains very limited.^12,14^

**Scheme 1:**
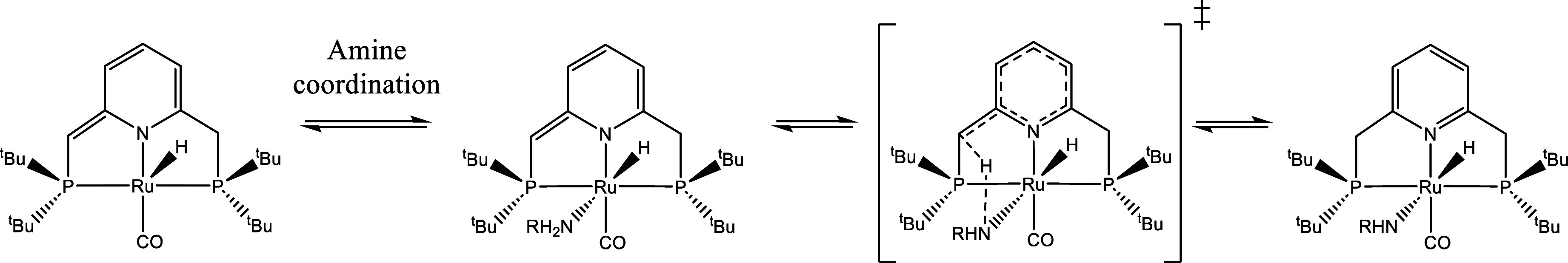
N–H Activation Reaction Studied in This Work

Theoretical studies in the field are also relatively scarce and have primarily focused on elucidating the reaction mechanism, the effect of the amine nature, the proposal of structural modifications in the ligands and the metal effect.^49,53–57^ It is generally accepted that N–H activation occurs through a single step and is significantly influenced by the nature of the amine. Specifically, for ammonia, an equilibrium that leans toward the reactants is observed. When electron-poor amines such as 4-nitroaniline and 2-chloro-4-nitroaniline are considered, the products are relatively favored. Conversely, for alkylamines with a stronger basic character (richer in electrons), such as isopropylamine, the opposite trend is observed.^47^ Besides, explicit calculations regarding the role of aromaticity related to bond activation in similar systems are relatively limited, with some few exceptions.^58–62^ One notable example is the work of Huang et al., who quantified the energy gains associated with system rearomatization during H–H activation using several Ru-based pincer ligands.^63^

In this contribution, we aim to deepen our understanding of the electronic factors influencing N–H activation through MLC, with the expectation that the findings reported herein will inspire the development of new systems with improved performance. For that, we have selected the original system proposed by Milstein (Scheme 1)^47^ with varied substrate scope and ligand modifications. In terms of methodology, we employ the quantum chemical topology (QCT),^64^ a theoretical framework that enables a chemically meaningful interpretation of the system’s properties in real space. Specifically, our focus is on the electron localization function (ELF) and the interacting quantum atom (IQA) approaches, which are briefly explained below.

The ELF enables the reconstruction of a system in terms of Lewis entities such as atoms, lone pairs, and covalent bonds. This fosters an intuitive interpretation of quantum mechanics within the context of chemistry.^65,66^ ELF’s utility extends beyond characterizing chemical bonds in static (or equilibrium) conditions, playing a significant role in studying reaction mechanisms. Indeed, bonding evolution theory (BET) integrates the topological analysis of the ELF along the intrinsic reaction coordinate (IRC) of a given chemical reaction with Thom’s catastrophe theory.^67^ This framework allows for the identification of the key chemical events, such as bond breaking/formation and the appearance/disappearance of lone pairs. This way, it facilitates the representation of electron flows using curly arrows, a fundamental pillar in modern chemistry.^68,69^ While the BET framework has been extensively applied to the understanding of a wide variety of organic reactions,^68–71^ its application to transition metal-based processes is considerably limited. One example consists on the elucidation of electron flows and the analysis of the chemoselectivity in the Noyori hydrogenation reaction.^72^ Regarding N–H activation, it is worth mentioning a study conducted by some of us in which we analyzed the electronic factors that determine the thermodynamics and kinetics of the oxidative addition of ammonia to a series of Ir­(I)-pincer promotors.^42^

Nevertheless, while the ELF offers a precise and visually appealing representation of electron pair movement during a chemical process, it falls short in depicting the energy aspects of it. Specifically, it lacks the ability to quantify the energy linked to a particular transformation or assess the strength of interactions among the chemical groups participating in the process. In light of this limitation, we combined BET with the IQA energy decomposition scheme.^73^ In our view, this methodology offers an important advantage over other alternatives, as it exclusively relies on the reduced first-order density matrix and the pair density. Consequently, they remain independent of any arbitrary reference state. Furthermore, IQA rigorously partitions the system’s energy into physically meaningful components and enables the decomposition of interactions between two atoms or groups of atoms. This decomposition results in a classical term (*V*_cl_), typically associated with the ionic or electrostatic contribution, and an exchange–correlation term (*V*_xc_), which is considered as the covalent counterpart of the interaction.^74^ In contrast to most energy decomposition analyses, IQA is independent of external references, and is therefore free from the many biases that the use of references lead to.^75^ Among several applications that have recently been reviewed elsewhere,^76,77^ IQA is especially relevant in the field of noncovalent interactions, for example, in the study of hydrogen,^78,79^ and halogen^80,81^ bonds, as well as in systems bearing metal atoms.^82–86^

IQA has also demonstrated to allow for a comprehensive understanding of chemically relevant processes, including, for example, the catalytic role of water molecules in the formation of sulfuric acid in acid rain,^78^ being also applied along the whole IRC of cycloaddition and peptide hydrolysis processes.^87–89^ IQA analyses in the field of transition metal-based chemical reactivity are relatively limited, but they have found some important applications in recent years. For instance, it has been used to investigate the C–H bond activation by Co­(III)-based complexes.^90^ Related studies by Handzlik et al. have explored the generation of propagating species using ruthenium-based Hoveyda–Grubbs-type initiators for olefin metathesis, using IQA to investigate noncovalent interactions between the metal and the ligand, as well as within the ligand scaffold.^91^

This work is organized as follows. We begin by outlining the methodology and criteria for model selection, followed by an analysis of ammonia fixation by the Ru­(II)–PNP complex developed by Milstein (Scheme 1). We then examine the influence of the amine substrate targeted for activation and, finally, propose rational modifications to the PNP-based ligand scaffold with the goal of enhancing the reaction performance and stimulating further experimental progress in the field.

## Results and Discussion

### Method Selection

As previously explained, we first considered the original Ru­(II)–PNP complex prepared by Milstein et al.^47^ To balance accuracy and computational effort, we substituted the original *tert*-butyl groups in the phosphines with methyl, yielding complex **1** (see Scheme 2). To validate this model, we also calculated the reaction and activation electronic and Gibbs energies for the original *tert*-butyl-substituted system across a diverse set of amines (*vide infra*). The results, obtained at the B3LYP-D3BJ/def2-TZVP level, are presented in Table S33 and Figures S2 and S3 of the Supporting Information. Overall, a strong correlation was observed (nearly linear with *R* ^2^ values above 0.89 for activation energies and 0.96 for reaction energies) between the outcomes of the model and those of the real system. This indicates that trends derived from the methyl-substituted model are reliable for predicting reaction behavior and for analyzing the electronic factors governing the process. Nonetheless, we acknowledge that results involving very bulky amines should be interpreted with caution, as steric hindrance is expected to play a significant role.

**Scheme 2:**
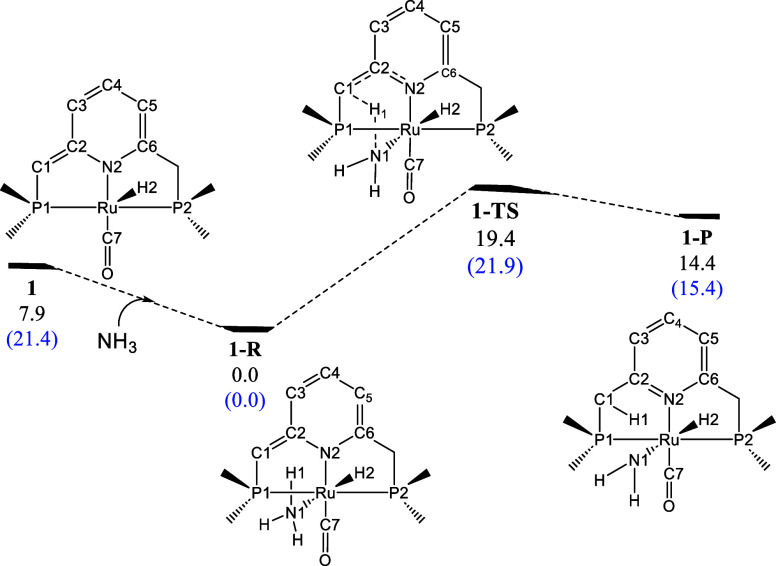
Reaction Profile for the Reaction of **1** with Ammonia Computed at the B3LYP-D3BJ Level of Theory^a^

a Gibbs energies are shown in black, and electronic energies in blue, both relative to **1-R** and in kcal·mol^–1^.

After assessing the validity of the catalyst modification, we tested several density functional approximations: B3LYP-D3BJ, M06, M06-L, M06-2X, and BP86-D3BJ for the reaction of **1** with ammonia, all in conjunction with the def2-TZVP basis set. It should be noted that B3LYP and the Minnesota family provided very similar results in terms of both reaction and activation Gibbs and electronic energies (Tables S1 and S2). The results are also comparable to those provided by BP86, although lower activation Gibbs energies were obtained in this case, i.e., 15.5 kcal·mol^–1^ vs 19.4 for B3LYP and 20.0 kcal·mol^–1^ for M06. This way, we opted by selecting B3LYP-D3BJ for further investigations, given that we have also applied it for previous calculations on ammonia fixation, BET, and IQA.^41–43,72,82,92^

We note that solvent effects were not included in the previous calculations. While the absence of solvation may influence the reaction energetics—particularly the amine coordination step—our primary aim is to analyze the electronic factors that govern the N–H activation process. For the calculation of topological descriptors and the establishment of general trends, we therefore consider gas-phase calculations to be sufficiently reliable. Moreover, as some of us have demonstrated elsewhere,^93^ the ionic/covalent energy partitioning provided by IQA allows to predict rather reliably the expected effect of solvents without the need to perform actual calculations.

### Reaction of **1** with Ammonia

The reaction profile of **1** with ammonia is provided in Scheme 2. Coordination of ammonia to the vacancy in **1** results in the formation of **1-R** (which serves as an energy reference). Subsequently, ammonia fixation via MLC occurs through transition state **1-TS**, requiring a Gibbs energy barrier of 19.4 kcal·mol^–1^. As a result, the product (**1-P**) is formed. However, the global process is thermodynamically unfavorable, exhibiting a change in Gibbs energy of 14.4 kcal·mol^–1^, which anticipates that there is an equilibrium between **1-R** and **1-P** that is highly displaced to **1-R**. These observations align with experimental findings and are consistent with the values reported by Milstein et al.^47^

The ELF for **1-R**, **1-TS**, and **1-P** is depicted in Figure 1. In **1-R**, the lone pair of ammonia coordinated to the metal center is represented by the disynaptic V­(Ru,N1) ELF basin, populated with 1.93 e^–^. At this point, we note that ELF basins are classified by their synaptic order, defined as the number of core basins (atoms) with which they are in contact; in this way, a disynaptic basin shares a boundary with two atoms (Ru and N in this case).

**Figure 1:**
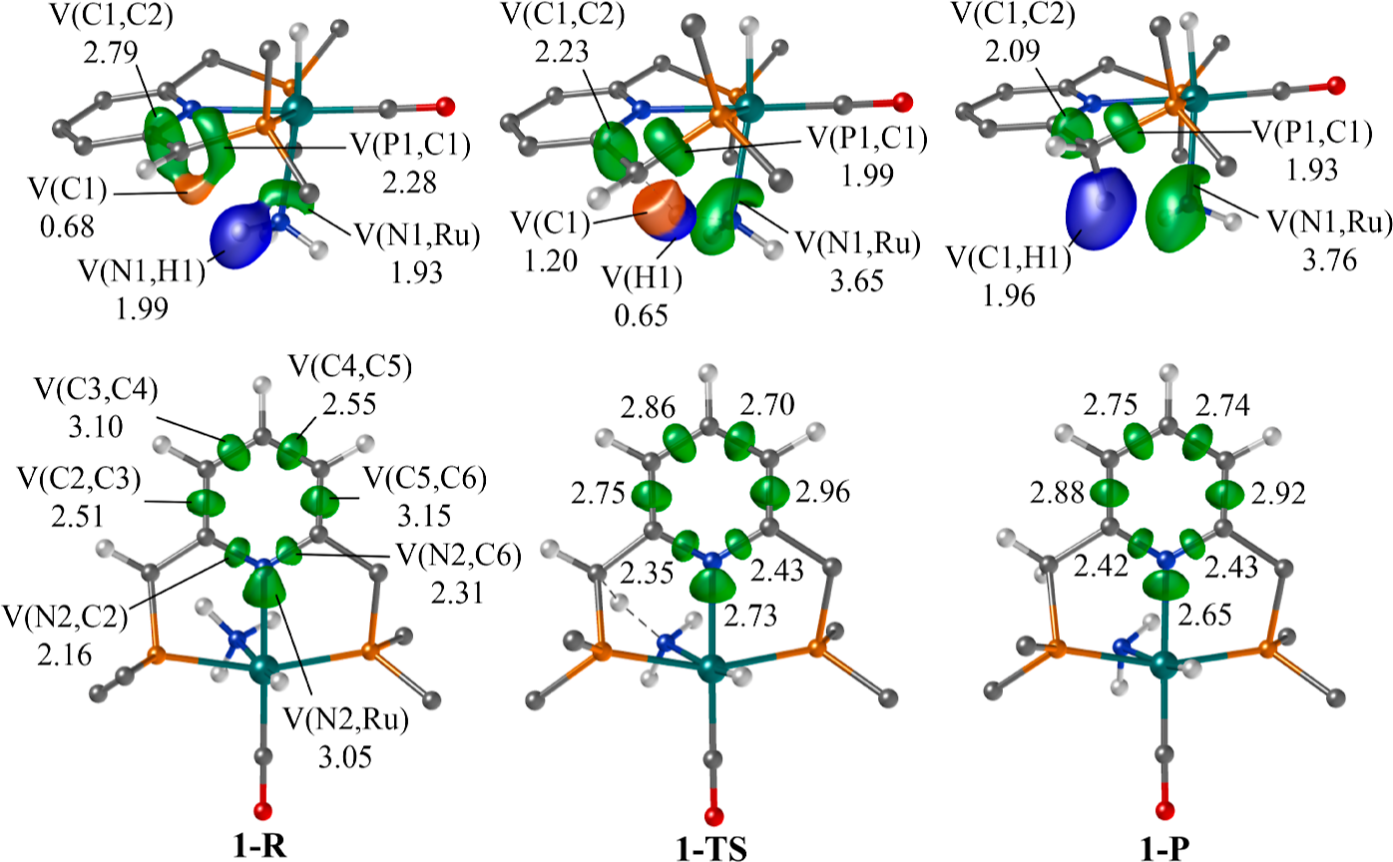
Representation of the main ELF basins (isovalue = 0.8) for **1-R**, **1-TS**, and **1-P**, along with their electron population (in electrons). In blue: hydrogenic basins, green: disynaptic basins, orange: monosynaptic basins (lone pairs). Many nonessential basins have been omitted for clarity. Atom nomenclature corresponds to that indicated in Scheme 2.

The N–H single bond character is also evident, as indicated by the hydrogenic V­(N1,H1) basin populations of 1.99 e^–^. Within the pincer PNP ligand, the unsaturated C1=C2 bond is represented by the disynaptic V­(C1,C2) basin, boasting a population of 2.79 e^–^. These findings suggest a partial double bond character, particularly considering significant delocalization along the pyridine-based scaffold. Notably, C1, which undergoes hydrogenation during the process, also features a monosynaptic basin, V­(C1), with a population of 0.68 e^–^. This attractor represents a pseudolone pair and plays a crucial role in the reaction mechanism, as we will elaborate below. The Ru center has a core basin C­(Ru) with a population of 13.25 e^–^, in agreement with an oxidation state of +II. Note in this regard that the basis set for Ru includes electron core potentials and only 16 valence electrons were explicitly considered.

The ELF basins within the dearomatized pyridine ring reveal the inequality among the various bonds. Namely, V­(C3,C4) and V­(C5,C6) show the highest populations among the C–C bonds, 3.10 and 3.15 e^–^, respectively, while V­(C2,C3) and V­(C4,C5) bear populations of 2.51 and 2.55 e^–^. Bond inequality is also observed for N2–C2 and N2–C6 (with populations of 2.16 and 2.31 e^–^, respectively). This is also supported by bond length differences (Table S3). This picture agrees with the (partially) dearomatized character of the ring.

In the transition state (**1-TS**), a new basin emerges due to the splitting of V­(N1,H1), that is, the breakage of ammonia’s N–H bond; giving rise to a hydrogenic basin, V­(H1), with a population of 0.65 e^–^. This result suggests that the hydrogen atom is mainly being transferred as a proton, as the basin population is significantly lower than 1 electron. The V­(Ru,N1) basin also undergoes relevant changes: its population increases from 1.93 to 3.65 e^–^. This shows that it takes up most of the population of the former V­(N1,H1) basin and leads to the formation of an amido group. The pseudo-lone pair basin, V­(C1), experiences a population increase from 0.68 to 1.20 e^–^. In general terms, these electrons come from the V­(C1,C2) and V­(P1,C1) basins, whose population decrease from 2.79 and 2.28 e^–^ to 2.23 and 1.99 e^–^, respectively.

Within the pyridinic ring there is a discernible trend toward bond equalization in terms of ELF basin electron population and bond lengths (see Figure 1 and Table S3 for detailed values). Overall, this suggests the triggering of aromatization within the ring. It is worth noting that the sums of the aforementioned basin populations in **1-R** and **1-TS** are nearly identical, indicating that these electron transfers occur within the ring itself. Besides, the population of the Ru core basin does not vary significantly, in agreement with the fact that its oxidation state does not change.

The main change in the formation of **1-P** is the creation of the V­(C1,H1) basin by combining the V­(C1) and V­(H1) basins. This feature signifies the formation of the C–H bond, with an electron population of 1.96 e^–^, consistent with a single bond. Simultaneously, the electron population in V­(C1,C2) and V­(C1,P1) decreases to 2.09 and 1.93 e^–^, in line with single C–C and C–P bonds. The V­(Ru,N1) basin increases up to of 3.76 e^–^, which supports the transfer of the NH_2_ fragment as an amido group.^46^ Also note that the process of rearomatization of pyridine reaches completion, in line with the equalization of the electron populations and distances in the C–C and C–N bonds. Similarly, C­(Ru) population keeps virtually constant (13.17 e^–^).

To attain a more detailed picture of the electron rearrangements that take place during the process, we performed a BET study. The evolution of the population of selected basins along the IRC is depicted in Figure 2, and their main characteristics (relative energies, selected distances and basin topology and populations) are provided in Table S3. Along the reaction path, three structure stability domains (SSDs) were identified, which correspond to the same obtained for **1-R** (SSD-I), **1-TS** (SSD-II), and **1-P** (SSD-III). The most relevant variations in basin populations correspond to the ones that have been previously explained for **1-R**, **1-TS**, and **1-P**. Thus, only the most relevant features of the BET profile are discussed.

**Figure 2:**
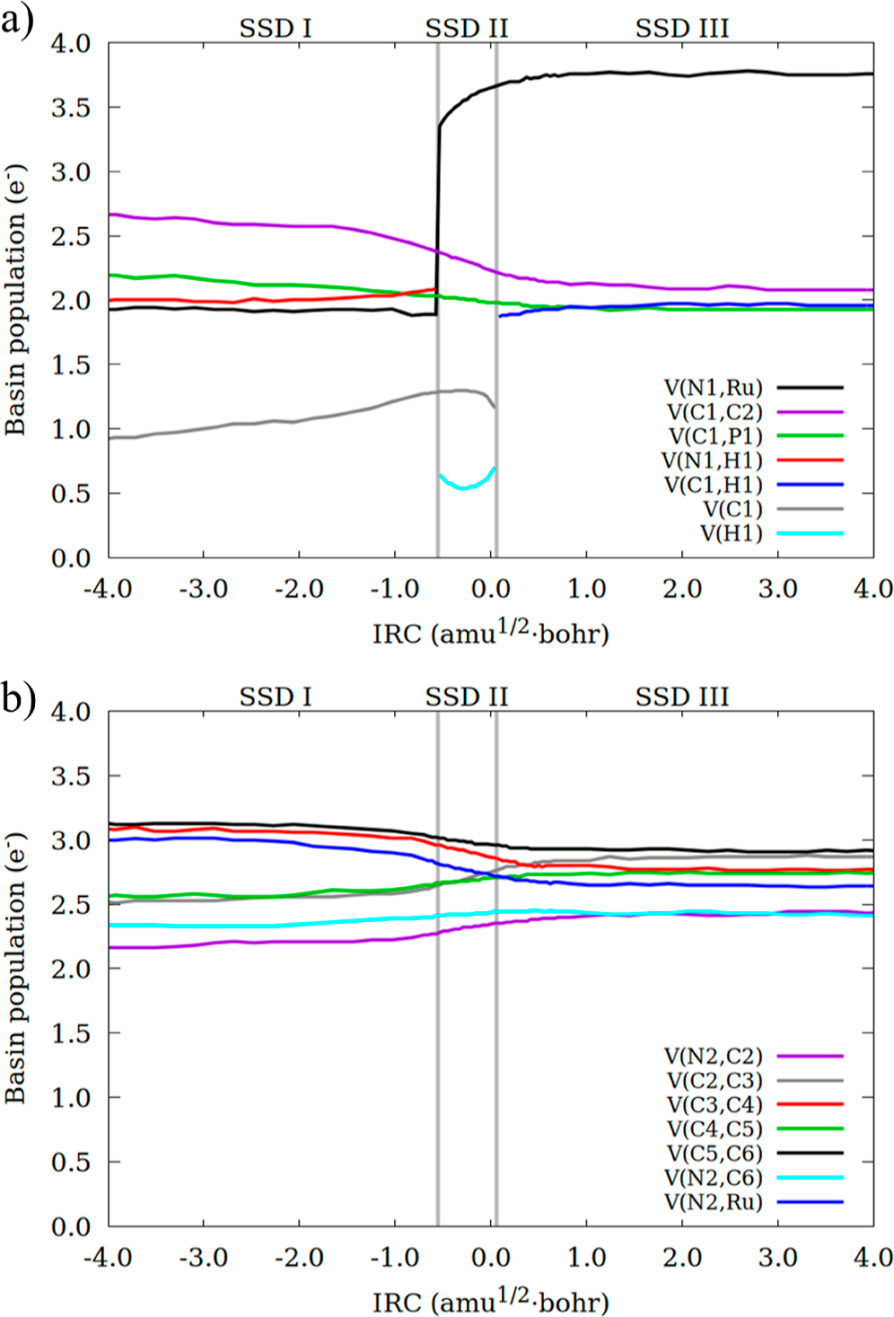
Integrated electron density (in electrons) for some ELF basins along the IRC path for the conversion of **1-R** to **1-P**. (a) Basins involving the metal center and ammonia, (b) basins in the pyridine-based ring. Bifurcation points separating the SSDs are indicated by vertical lines.

Along SSD-I, V­(C1) and V­(N1,H1) increase their populations in 0.57 e^–^ and 0.10 e^–^, respectively, while V­(C1,C2) and V­(C1,P1) undertake a decrease in population of 0.39 and 0.26 e^–^. These variations provide a neutral balance, which is also observed in the dearomatized pyridine ring (Figure 2b). Nonetheless there are non-negligible electron flows (0.10, 0.22 e^–^) within basins, as previously explained. Note that, while the electron flow from V­(C1,C2) and V­(C1,P1) to V­(C1) and V­(N1,H1) occurs throughout the entire SSD, the variation in the dearomatized ring is observed in the very last part. SSD-I concludes with the conversion of the V­(N1,H1) basin into V­(H1).

SSD-II is the narrowest and initiates with a sudden increase in the population of the V­(Ru,N1) basin, which absorbs 1.46 e^–^ (out of 2.09 e^–^) from the former V­(N1,H1) basin. The remaining electrons are transferred to V­(H1) basin. As we move through the domain, the population of the V­(Ru,N1) basin continues to rise, reaching 3.66 e^–^, while that of V­(H1) increments up to 0.70 e^–^. Such population growth comes at the expense of V­(C1,C2), V­(C1,P1), and V­(C1). The electron flow is completed by further redistribution of electrons within the pyridinic ring. Note that in the initial segment of SSD-II the population of V­(H1) declines from 0.64 to 0.51 e^–^, but it rebounds and increases once we move into the latter part of the domain. At the same time, in the first part, the V­(C1) basin maintains a steady population at around 1.3 e^–^ that decreases in the latter part to 1.16 e^–^. Finally, these two basins combine, giving rise to V­(C1,H1).

In SSD-III, the final electron rearrangements occur: the V­(C1,H1) basin increases to 1.96 e^–^, consistent with a single C–H bond. Concurrently, the V­(Ru,N1) basin rises slightly to 3.75 e^–^, reflecting the formation of an amidic nitrogen. Minor electron redistributions within the pyridinic scaffold (≤0.10 e^–^) toward bond equalization further indicate the restoration of aromaticity. Note that variations in basin populations predominantly occur within the very first part of the domain, with the remainder part being dedicated to the final geometrical adjustments.

The energetic counterpart of the analysis is provided by the IQA framework. Given the high computational cost of such calculations, we only considered **1-R**, **1-TS**, and **1-P**. In systems with constant charge, the classical electrostatic interaction (*V*_cl_) between multiple atoms tends to cancel out, making the exchange–correlation contribution (*V*_xc_) more significant for describing chemical bonding.^74^ For electron reorganization within the dearomatized pyridine moiety, which maintains a neutral electron-flow between centers, we report only *V*_xc_ (see Table S4 for additional contributions). For interactions involving H1, C1 and N1 with a significant charge variation, the electrostatic contribution (*V*_cl_), and *E*_int_, are also explained.

The N1–H1 interaction decreases from *E*_int_ = −254.2 kcal·mol^–1^ in **1-R** to −142.8 kcal·mol^–1^ in **1-TS** and −24.0 kcal·mol^–1^ in **1-P** (Table 1). This correlates with the bond breaking and subsequent sharp decrease in the strength of *V*_xc_ from −152.3 kcal·mol^–1^ in **1-R** to −55.4 kcal·mol^–1^ in **1-TS**, being almost negligible in **1-P** (−6.7 kcal·mol^–1^). As anticipated, the *V*_cl_ interaction is significant, and, given the opposite signs of the atomic charge in H1 (positive) and N1 (negative), it takes negative (attractive) values (see Table S5 for QTAIM charges).

**Table 1:** *E*_int_, *V*_xc_, and *V*_cl_ for Selected Interactions in **1-R**, **1-TS**, and **1-P**

	*E*_int_ (kcal·mol^–1^)			*V*_xc_ (kcal·mol^–1^)			*V*_cl_ (kcal·mol^–1^)		
	1-R	1-TS	1-P	1-R	1-TS	1-P	1-R	1-TS	1-P
N1–H1	–254.2	–142.8	–24.0	–152.3	–55.4	–6.7	–101.9	–87.3	–17.4
C1–H1	–50.5	–156.2	–162.9	–4.3	–89.7	–165.8	–46.3	–66.5	2.9
Ru–N1	–170.3	–202.0	–213.7	–60.4	–76.0	–86.8	–110.0	–125.9	–127.0
P1–C1	–629.8	–527.7	–436.7	–144.9	–133.3	–125.0	–484.8	–394.3	–311.7
C1–C2	–282.3	–248.6	–217.1	–261.5	–218.2	–196.9	–20.8	–30.4	–20.3

The C1–H1 interaction follows an opposite evolution, *E*_int_ increasing from −50.5 kcal·mol^–1^ in **1-R** to −156.2 kcal·mol^–1^ in **1-TS**, and −162.9 kcal·mol^–1^ in **1-P**, in agreement with the formation of a single C–H bond. As expected, *V*_xc_ increases from **1-R** (−4.3 kcal·mol^–1^), in which there is barely any electron sharing between C1 and H1 to **1-P** (−165.8 kcal·mol^–1^). Interestingly, the highest (more stabilizing) *V*_cl_ term is reached at **1-TS** (−66.5 kcal·mol^–1^). This feature can be rationalized by considering that, at the zeroth order, *V*_cl_ can be approximated as *q*_1_·*q*_2_/*r*_12_. In this way, charges in **1-R** are approximately the same as those in **1-TS** (see Table S5), while the C1–H1 distance in the latter is much shorter (2.757 Å in **1-R**, 1.314 Å in **1-TS**). The formation of C1–H1 bond also affects C1 interactions with C2 and P1. Namely, the formal C1–C2 bond order decrease from (partially) double to single translates into an important decrease in *V*_xc_ (from −261.5 to −196.6 kcal·mol^–1^), a similar behavior being observed for C1–P1 bond.

The Ru–N1 interaction warrants special attention. The *V*_xc_ term increases very significantly from **1-R** to **1-P** (from −60.4 to −86.8 kcal·mol^–1^) due to the enhanced electron-sharing interactions between the two centers, as revealed by the ELF analysis. Additionally, *V*_cl_ becomes more favorable (from −110.0 to −127.0 kcal·mol^–1^) due to the shortening of Ru–N1 distance, as N1 and Ru remain essentially unchanged (Table S5). Since both components contribute to the same direction, the total interaction energy (*E*_int_) is more favorable in **1-P** (−213.7 kcal·mol^–1^) than in **1-R** (−170.3 kcal·mol^–1^).

The rearomatization of the pyridine-based ligand is also unraveled by IQA analysis, which is discussed based on *V*_xc_ (Table 2). As we can see at first glance, the *V*_xc_ values evolve toward symmetrical equalization. Also note that the *V*_xc_ values in the transition state are closer to those of the product than to those of the reactant, which agrees with the fact that ELF changes in SSD-III are minor compared to SSD-I and II.

**Table 2:** *V*_xc_ for Selected Interactions in the Conversion of **1-R** to **1-P**

	*V*_xc_ (kcal·mol^–1^)		
	1-R	1-TS	1-P
C2–C3	–227.8	–244.9	–253.5
C3–C4	–280.6	–266.5	–260.9
C4–C5	–243.5	–255.0	–259.9
C5–C6	–264.0	–256.9	–254.8
N2–C2	–215.7	–225.5	–232.2
N2–C6	–233.3	–234.0	–233.7
Ru–N2	–79.0	–74.4	–73.8

While significant changes occur within the pyridine ring, the *V*_xc_ interaction of Ru with P1 P2, H2 (the hydride ligand), and C7 (the C atom belonging to CO ligand), only show minor variations (see Table S4).

### Effect of the Amine

We then investigated the effect of the substituent groups in the substrate by replacing ammonia with a primary amine. To enhance clarity, we categorized the amines into two groups: those with electron-donating groups (EDG) and those with electron-withdrawing groups (EWG) and selected 18 different amines to represent both categories (Chart S1). As expected, based on previous results, EDG systems (such as alkyl substituents) show a worse performance than ammonia, in both kinetics (higher activation Gibbs energies) and thermodynamics (more positive reaction Gibbs energy). On the contrary, systems bearing EWG, that is, more acidic amines, exhibit lower activation and reaction Gibbs energies than ammonia, as shown in Table S6.^47,53^ On passing, we note that the energetics of the amine coordination to **1** exhibit notable variations depending on the substituents, consistent with reports by Milstein et al.^47^ Amines with EWGs tend to relatively disfavor coordination. Nonetheless, our calculations indicate that the coordination process remains thermodynamically favorable, albeit marginally so in some cases—such as with 2,4-bis­(trifluoromethyl)­aniline (2.2 kcal·mol^–1^). This contrasts with Milstein calculations, suggesting unfavorable coordination for certain systems. We attribute this discrepancy to differences in computational methodology (effect of the functional, basis set, and solvation model); however, conclusions drained by both studies are consistent with each other.

For analyzing the electron flows and atomic interactions within the process, and identify potential variations with respect to ammonia—substrate dependent chemical electron flow sequences have been unraveled by BET for other processes^72^—we considered a representative example of each kind of amine: isopropylamine (propan-2-amine, ^i^Pr-NH_2_) and 2,4-bis­(trifluoromethyl)­aniline (2,4-(CF_3_)_2_-Ph-NH_2_) as EDG and EWG cases, respectively. The Gibbs energy results for the reaction of NH_3_, ^i^PrNH_2_ and 2,4-(CF_3_)_2_-Ph-NH_2_ with **1** are provided in Scheme 3. Given that we are now changing the amine, we refer to the reactant complex, the transition state, and the product as **1-R′**, **1-TS**’, and **1-P′**, respectively. We note that additional rearrangements have been reported for isopropylamine, leading to H_2_ elimination and isopropylimine formation.^47^ Nonetheless, we consider ^i^Pr–NH_2_ to be an appropriate model for assessing the influence of substrate substituents with an electron-donating character.

**Scheme 3:**
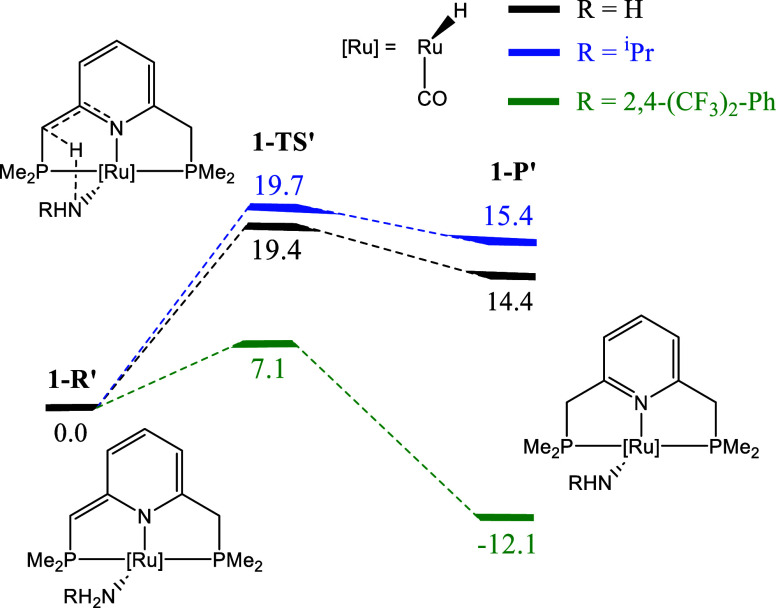
Reaction Profile (Relative Gibbs Energy in kcal·mol^–1^) Relative to **1-R′** for the Reaction of **1** with Ammonia (Black Lines), Isopropylamine (Blue Lines), and 2,4-Bis­(trifluoromethyl)­aniline (Green Lines)

The BET results for selected basins for ^i^Pr-NH_2_ and 2,4-(CF_3_)_2_-Ph-NH_2_ are provided in Figure 3. Noteworthy, the same SSDs are observed in all cases, which reveals that the amine does not alter the main chemical events that take place along the reaction pathway. In addition, the C­(Ru) basin population does not change, pointing toward the Ru­(II) oxidation state not being affected by the amine. The evolution of basin populations within the pyridine-based ring is virtually the same for ^i^Pr-NH_2_, 2,4-(CF_3_)_2_-Ph-NH_2_, and ammonia, and is provided in Figure S1.

**Figure 3:**
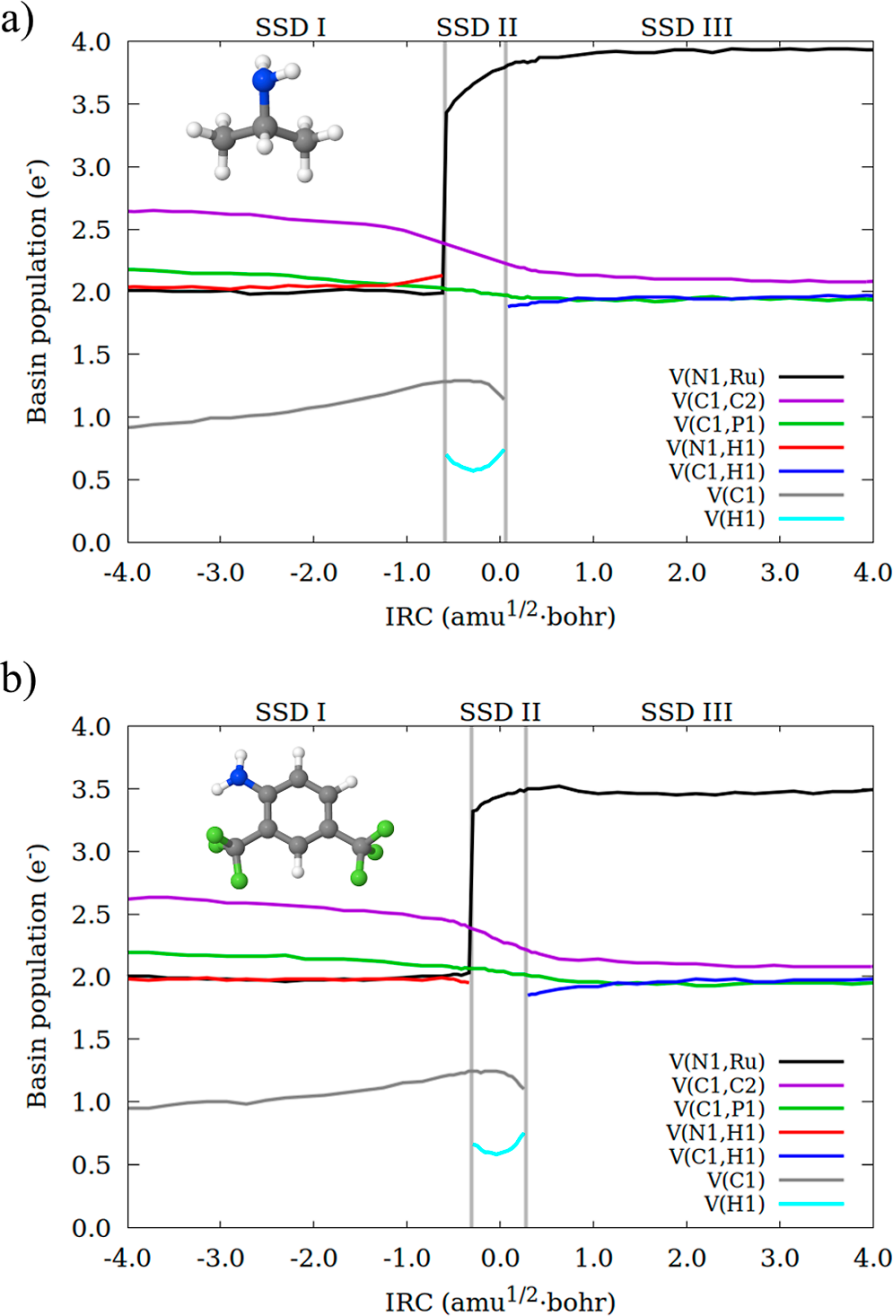
Integrated electron density (in electrons) for some ELF basins along the IRC path for the conversion of **1-R′** to **1-P′** with (a) isopropylamine, (b) 2,4-bis­(trifluoromethyl)­aniline. Bifurcation points separating the SSDs are indicated by vertical lines.

The main variations in SSD-I are found in V­(N1,H1) and V­(Ru,N1). The initial populations are higher for ^i^Pr-NH_2_ (EDG, 2.03 and 2.00 e^–^, respectively) than for NH_3_ (1.99 and 1.93 e^–^) and 2,4-(CF_3_)_2_-Ph-NH_2_ (EWG, 1.97 and 1.99 e^–^). The population of the V­(N1,H1) basin in NH_3_ and ^i^Pr-NH_2_ increases along SSD-I (in 0.10 e^–^) at the expense of V­(Ru,N1). Contrarily, variations in 2,4-(CF_3_)_2_-Ph-NH_2_ are very small, and it is the V­(N1,H1) that slightly decreases in population (by only 0.02 e^–^) and the V­(Ru,N2) that increases (in 0.04 e^–^).

At the beginning of SSD-II, the initial populations of the V­(Ru,N1) basin are not too dissimilar, while being higher for ^i^Pr-NH_2_ (3.43 e^–^) than for NH_3_ and 2,4-(CF_3_)_2_-Ph-NH_2_ (3.35 and 3.32 e^–^, respectively). Nonetheless, their evolution is quite different: for ^i^PrNH_2_ it reaches a value of 3.79 e^–^ at the end of the domain, while such a population is lower for ammonia (3.66 e^–^) and 2,4-(CF_3_)_2_-Ph-NH_2_ (3.48 e^–^). This result correlates with the electron donor character of isopropyl, which favors electron density accumulation on the N lone pair. Along SSD-III the population of V­(Ru,N1) exhibits important differences: for ^i^Pr-NH_2_, it increases up to 3.95 e^–^, which is notably higher than that of NH_3_ (3.75 e^–^) and 2,4-(CF_3_)_2_-Ph-NH_2_ (3.47 e^–^), this being the main difference between the three systems. The rearomatization processes are virtually equal for the three cases (Figure S1).

IQA results for selected interactions are summarized in Table 3. Due to the previously discussed compensation of differences in *V*_cl_ across the whole molecule, the comparative analysis is performed in terms of *V*_xc_, all the contributions being provided in Tables S9 and S10.

**Table 3:** *V*_xc_ for Selected Interactions in the Conversion of **1-R′** to **1-P′** with Isopropylamine and 2,4-Bis­(trifluoromethyl)­aniline

		*V*_xc_ (kcal·mol^–1^)		
		1-R′	1-TS′	1-P′
	N1–H1	–156.4	–53.6	–7.1
^i^Pr-NH_2_	C1–H1	–2.8	–92.7	–165.5
	Ru–N1	–60.8	–76.1	–86.4
2,4-(CF_3_)_2_-Ph-NH_2_	N1–H1	–143.9	–71.7	–2.1
	C1–H1	–6.9	–69.0	–168.9
	Ru–N1	–48.2	–59.5	–77.9

As anticipated, the N1–H1 interaction energetics in **1-R′** (i.e., before bond activation) strongly depends on the amine, in agreement with the ELF populations. *V*_xc_ is more favorable for ^i^Pr-NH_2_ (−156.4 kcal·mol^–1^) than for ammonia (−152.3 kcal·mol^–1^) and 2,4-(CF_3_)_2_-Ph-NH_2_ (−143.9 kcal·mol^–1^). The values are consistent with the nature of the amine, which ultimately has an effect on the energy profiles. In this regard, in **1-TS′**, the most favorable *V*_xc_ is found in 2,4-(CF_3_)_2_-Ph-NH_2_, which again is translated in the lowest activation energy.

The C1–H1 interaction exhibits the opposite behavior. As for ammonia, *V*_xc_ is nearly negligible in **1-R′**. In **1-TS′**, it is significantly more favorable for ^i^Pr-NH_2_ (−92.7 kcal·mol^–1^) than for 2,4-(CF_3_)_2_-Ph-NH_2_ (−69.0 kcal·mol^–1^), taking a value that is very close to that of ammonia (−89.7 kcal·mol^–1^). Contrarily, the final interaction value (in **1-P′**) is very similar in all cases, as expected given the single C1–H1 bond that is formed. The Ru–N1 interaction also undertakes significant differences. *V*_xc_ is more favorable for ^i^Pr-NH_2_ (in **1-R′**, **1-TS′** and **1-P′**), and increases from **1-R′** to **1-P′**, in line with the higher population accumulated on the V­(Ru,N1) basin.

The previous results indicate that 2,4-(CF_3_)_2_-Ph-NH_2_ comparatively facilitates N1–H1 bond cleavage, whereas the C1–H1 interaction is favored for ^i^Pr-NH_2_. Although the impact on the Ru–N1 interaction is less pronounced, it is more favorable for 2,4-(CF_3_)_2_-Ph-NH_2_. The variation in the sum of these interactions when moving from **1-R′** to **1-P′** is −39.0 kcal·mol^–1^ for ^i^Pr-NH_2_, compared to −49.9 kcal·mol^–1^ for 2,4-(CF_3_)_2_-Ph-NH_2_, in agreement with the significantly greater stability of the latter. Nonetheless, variations from **1-R′** to **1-TS′** are −2.4 kcal·mol^–1^ for ^i^Pr-NH_2_ and −1.2 kcal·mol^–1^ for 2,4-(CF_3_)_2_-Ph-NH_2_, which does not account for the significantly different activation energies observed in these systems. This suggests that additional interactions must be taken into account. In particular, the interaction between the nitrogen atom and the substituent (R), which involves several atoms, had not been considered in the previous analysis. Given the differing nature of the substituent groups, analyzing the relative changes in the NH–R interaction provides more meaningful insight. To address this, we partitioned the system into five fragments, including the NH–R unit, as shown in Scheme 4. Interaction values between selected fragments are presented in Table 4, with the full data set and individual contributions available in Tables S13–S15.

**Scheme 4:**
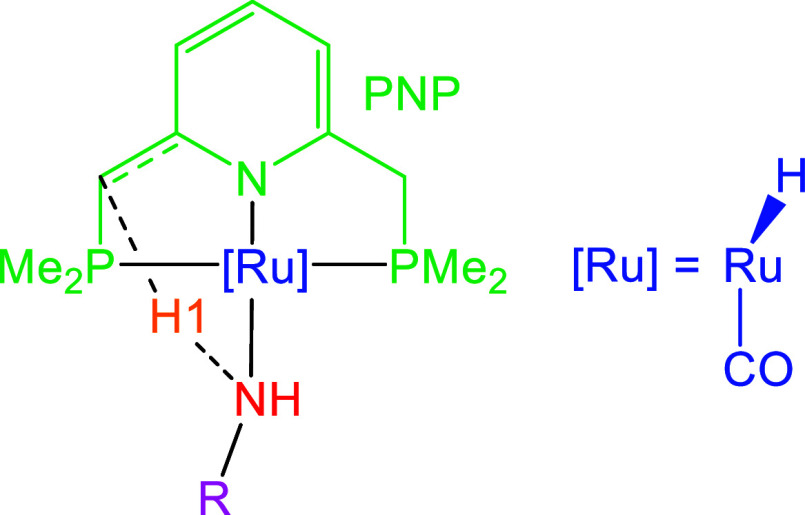
Schematic Representation of the Five Groups Considered in the IQA Analysis

**Table 4:** *V*_xc_ for Selected Interactions Between Groups in the Conversion of **1-R′** to **1-P′** with Isopropylamine, Ammonia, and 2,4-Bis­(trifluoromethyl)­aniline

		*V*_xc_ (kcal·mol^–1^)		
		1-R′	1-TS′	1-P′
	NH–R	–209.3	–225.2	–232.8
^i^Pr-NH_2_	NH–H1	–157.6	–54.1	–7.2
	PNP–H1	–5.4	–102.2	–177.8
NH_3_	NH–R	–163.2	–170.4	–174.2
	NH–H1	–153.4	–55.9	–6.8
	PNP–H1	–7.4	–99.3	–178.2
2,4-(CF_3_)_2_-Ph-NH_2_	NH–R	–234.9	–257.4	–288.7
	NH–H1	–144.8	–72.4	–2.3
	PNP–H1	–11.0	–80.0	–181.3

Notably, the NH–R interaction becomes significantly more stabilizing in 2,4-(CF_3_)_2_-Ph-NH_2_, increasing by −22.5 kcal·mol^–1^ from **1-R′** to **1-TS′**, and by −53.8 kcal·mol^–1^ from **1-R′** to **1-P′**. In contrast, for ammonia and ^i^Pr-NH_2_, the changes are markedly smaller, in agreement with their higher reaction barrier.

Consistent with previous results for the C1–H1 and N1–H1 interactions, a comparison between NH–H1 and PNP–H1—that is, the interaction between the transferred hydrogen atom and the NH or PNP ligand scaffold (depicted in green in Scheme 4)—shows that the overall valence contribution is nearly neutral. As a result, the variation in *V*_xc_ is governed by the NH–R interaction.

All in all, our results suggest that more acidic systems—such as those bearing EWG (EWGs), e.g., 2,4-bis­(trifluoromethyl)­aniline—exhibit a greater increase in exchange–correlation interaction energy between the substituent and the NH fragment upon reaction, comparatively stabilizing both the transition state and the product. This trend aligns with the ELF analysis, which shows that the nitrogen lone pair has a lower electron population in EWG-containing systems, reflecting greater delocalization toward the R group and correlating with stronger interaction energies.

### Structural Modifications of Complex **1**

Below, we analyze the effects of structural modifications on the ligand scaffold. From the preceding analysis, as well as previous reports,^49^ we conclude that the H atom of the N–H bond is transferred in a chemical state akin to a proton. Consequently, ligand functionalities that stabilize the proton should facilitate the reaction. In this context, the pyridine-based ligand scaffold in **1** adopts a resonance form that places a lone pair on C1—the site of hydrogenation—as illustrated in Scheme 5, consistent with the presence of a monosynaptic V­(C1) basin at this atom. Accordingly, we propose that electron-donating substituents on the ring would favor this resonance structure by relatively stabilizing the formal positive charge on the pyridine framework, leading to an enhanced electron density at C1 and, consequently, facilitating the reaction. In this line, other authors have reported that substitutions in the pyridine-based ring significantly impact system aromaticity and have leveraged these effects for ligand design in closely related systems.^13,61,63^

**Scheme 5:**
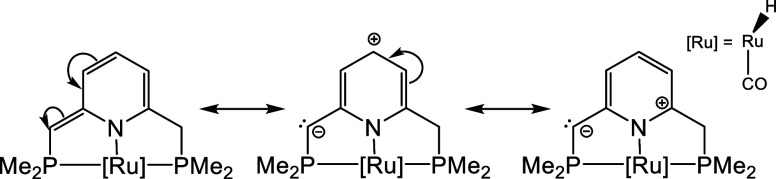
Schematic Representation of Some of the Resonance Forms that Take Place within the Pyridine-Based Ligand Scaffold

We examined a series of modifications incorporating various EDGs and EWGs in the ligand (Chart S2) and analyzed its effect on reaction kinetics (activation energy) and thermodynamics (Δ*G* of reaction). For these studies we consider ammonia as the substrate. As anticipated, EDG favored both the kinetics and thermodynamics of the reaction (Tables S16 and S17). In the following, two case examples are explained in detail (Scheme 6). Specifically, we considered two different X substituents in the 2 and 4 positions of the pyridine-based scaffold: X = Cl, as EWG; and X = NH_2_, as resonance EDG. For the sake of clarity, we refer to the complex, reactant, transition state and product as **1­[X]**, **1-R­[X]**, **1-TS­[X]**, and **1-P­[X]**, respectively. Note that X = Cl led to an increase in the activation and reaction Gibbs energies with respect to the initial complex, **1**, of 2.1 and 2.8 kcal·mol^–1^, respectively. On the contrary, X = NH_2_ translated into a decrease in the activation and reaction Gibbs energy of 2.8 and 5.1 kcal·mol^–1^, respectively.

**Scheme 6:**
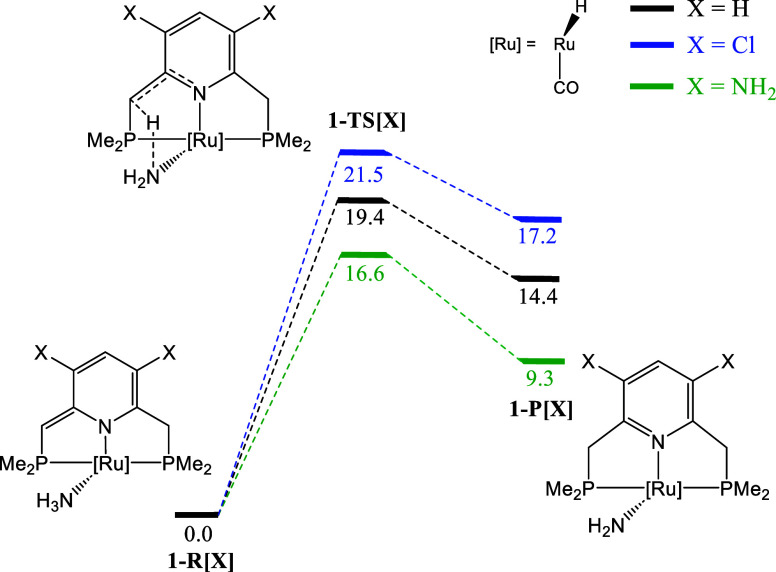
Reaction Profile for the Reaction of **1-R­[X]** with Ammonia. Relative Gibbs Energy Values are Given in kcal·mol^–1^

The BET results for selected basins for the reaction of **1­[NH**_**2**_ **]** and **1­[Cl]** with ammonia are provided in Figure 4 (see Tables S18–S22 for the additional basins and systems). In both cases, the same SSDs are found, which indicates that the proposed modifications do not change the main chemical events that take place along the N–H activation process.

**Figure 4:**
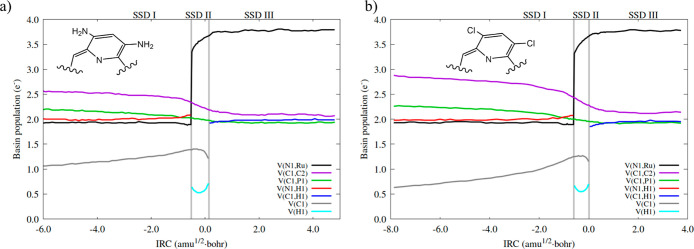
Integrated electron density (in electrons) for some ELF basins along the IRC path for the reaction of ammonia with (a) **1­[NH**_**2**_ **]** and (b) **1­[Cl]**.

Interestingly, the change in population of many relevant basins for the process, such as V­(H1), V­(C1,H1), and V­(Ru,N1) is barely affected by the modifications in **1**. Contrarily, there are significant variations in V­(C1), V­(C1,C2), and, to a lesser extent, V­(C1,P1). Indeed, at the start of SSD-I, the population of V­(C1) basin is 1.05 e^–^ in **1­[NH**_**2**_ **]** and 0.63 e^–^ in **1­[Cl]**, while the population of V­(C1,C2) basins are 2.57 e^–^ and 2.88 e^–^, respectively. This agrees with our initial analysis based on resonance structures (Scheme 5), which points to a higher electron density accumulated at V­(C1) in the case of **1­[NH**_**2**_ **]**. This is further supported by the QTAIM charges of C1 in **1-R­[X]**, which are more negative for X = NH_2_ (Tables S28 and S29). The difference between V­(C1) and V­(C1,C2) basins remains significant along the whole domain, but it tends to reduce along it, in such a way that the final populations for V­(C1) and V­(C1,C2) are 1.39 and 2.39 e^–^, respectively, for **1­[NH**_**2**_ **]** and 1.25 and 2.43 e^–^ for **1­[Cl]**.

The difference in V­(C1) and V­(C1,C2) is much lower in SSD-II. For instance, V­(C1) takes values of 1.21 and 1.15 e^–^ at the end of such a domain for **1­[NH**_**2**_ **]** and **1­[Cl]**, respectively. The formation of the C–H bond in SSD-III resonates with the equalization tendency, in such a way that the initial population of the V­(C1,H1) basin is slightly higher for **1­[NH**_**2**_ **]** (1.92 e^–^) than for **1­[Cl]** (1.85 e^–^), respectively. The difference decreases even more when reaching the final product at the end of the domain (1.99 and 1.95 e^–^, respectively), in agreement with the formation of a single C–H bond.

At this point, it should be noted that the change on the X group also leads to relevant changes in the populations of the V­(C,C) and V­(C,N) basins related to the pyridine-based ring (Tables S18–S22). Indeed, the most affected basin is V­(C1,C2) which, in **1-R′** bear significantly higher populations (about 0.3 e^–^) for X = Cl, in correlation with the lower population of the V­(C1) basin in such system. Although, to a lower extent, this effect is also observed in most basins constituting the dearomatized pyridinic ring, with population differences of about 0.1 e^–^. This difference is reduced along the reaction pathway, the final basin populations in **1-P′** being nearly equal. Note that providing an exhaustive analysis of electron redistribution within the ligand scaffold is beyond the scope of this contribution, which focuses on N–H activation. Nonetheless, all relevant data is available in the Supporting Information for interested readers (Tables S18–S22).

Table 5 collects *V*_xc_ values for N1–H1, C1–H1, and C1–C2 in **1­[NH**_**2**_ **]** and **1­[Cl]** systems, which we identify as the key ones for the process (see Tables S23 and S24 for the complete set of values). Taking *V*_xc_ for the C1–C2 bond as a proxy for low aromatization of the pyridinic ring, the **1-R­[NH**_**2**_ **]** reactant is already seen to display a considerably smaller double bond character than that of **1-R­[Cl]**. The initial 14.9 kcal·mol^–1^ difference between both decreases to about 6 kcal·mol^–1^ in **1-TS** and becomes much smaller (1.8 kcal·mol^–1^) in the product, also in agreement with BET insights. Therefore, preorganizing the reactant’s electronic structure may play a relevant role. Actually, these three key interactions alone reasonably well predict the reaction profile. By adding the N1–H1, C1–H1, and C1–C2 *V*_xc_ contributions, one can easily check that if we take **1-R** as a reference, there is a 7.7 and 7.5 kcal·mol^–1^ energy difference at **1-TS** and **1-P** states, respectively, favoring the **1­[NH**_**2**_ **]** over the **1­[Cl]** system. The IQA perspective thus allows us to pinpoint the N1–H1–C1–C2 region as key in determining the effects of substitution in the pyridinic ring.

**Table 5:** *V*_xc_ for Selected Interactions in the Reaction of **1­[NH**_**2**_ **]** and **1­[Cl]** with Ammonia

		*V*_xc_ (kcal·mol^–1^)		
		1-R[X]	1-TS[X]	1-P[X]
**1[NH**_**2**_ **]**	N1–H1	–150.6	–58.9	–4.4
	C1–H1	–5.3	–85.6	–169.1
	C1–C2	–251.4	–212.9	–195.3
**1[Cl]**	N1–H1	–153.5	–53.3	–6.4
	C1–H1	–3.5	–92.5	–165.1
	C1–C2	–266.3	–218.9	–197.1

## Conclusions

In this study, we applied state-of-the-art computational methods within the QCT framework to explore, in real space, the electronic factors influencing the kinetics and thermodynamics of N–H activation in a seminal Ru­(II)–PNP complex that operates via an MLC dearomatization/rearomatization mechanism. The study is based on the premise that the successful design of new organometallic complexes with enhanced properties requires a detailed understanding of the electronic effects governing their reactivity.

The BET results indicate that activation of the N–H bond in ammonia by Ru­(II)–PNP complex **1** occurs through a process involving three distinct structural stability domains. First (from SSD-I to II), the N–H bond breaks, leading to the formation of a monosynaptic V­(H) basin with a population lower than 1 e^–^, which resonates with the H atom being transferred as a proton. Besides, a Ru–NH_2_ *amido* group is formed. Then (from SSD-II to III), the proton bonds to the pseudo-lone pair in the C atom of the ligand arm (merging of V­(C) and V­(H) basins to form a disynaptic V­(C,H) one), forming a single C–H bond. Such a process is coupled to the rearomatization of the pyridine-based ring in the PNP ligand, which is more intense in SSD-II, starting in the final part of SSD-I and finishing in the beginning of SSD-III. IQA energy decomposition scheme correlates with these results, indicating that the strength of C–C and C–N bonds (measured by *V*_xc_ term) equalize along the rearomatization process.

Modifying the amine substituents significantly impacts the reaction’s kinetics and thermodynamics, with EWG enhancing both. This trend aligns with the transfer of the N–H hydrogen as a proton, as EWGs increase amine acidity, promoting H release and facilitating *amido* product formation. However, the change in the amine substituents does not alter the molecular reaction mechanism, which is defined by the same ELF SSDs. Besides, the rearomatization process of the pyridine-based ligand is barely affected by the substrate changed. On the contrary N–H, C–H, and NH-R interactions experience important differences.

Several potential modifications to the PNP ligand have been proposed for ligand design, focusing on altering the substituents at the 2 and 4 positions of the dearomatized pyridine-based rest. Electron-donor groups, such as –NH_2_, favor charge accumulation at the C atom situated in the ligand arm that is to be hydrogenated. Since the H atom is transferred as a proton, this leads to improved system performance. Beyond advancing the fundamental understanding of the process, we hope this finding inspires experimentalists and aids in designing new systems with enhanced activity.

## Computational Details

Electronic structure calculations were performed at the Density Functional Theory (DFT) level in the gas-phase. We considered B3LYP,^94^ M06,^95^ M06-L,^96^ M06–2X,^95^ and BP86 ^97^ exchange–correlation functionals. We combined B3LYP and BP86, with D3BJ empirical correction dispersion scheme.^98^ Geometry optimizations were performed by using the Gaussian 16,^99^ and triple-ζ def2-TZVP basis sets.^100^ Gibbs energies were obtained at 298.15 K and 1 atm, according to the harmonic rigid rotor approximation. For calculating the wave functions for IQA calculations, we performed single point calculations on the optimized structures, by using the zeroth order regular approximation to take into account relativistic effects in Ru centers.^101^ In the former case, we considered the SARC-ZORA-TZVP basis set for, and the SARC/J auxiliary basis sets^102^ together with the RIJCOSX approximation to accelerate the calculations;^103^ as implemented in the Orca 5.0 software package.^104^

ELF calculations were carried out by using TopMod package,^105^ using the corresponding B3LYP monodeterminantal all-electron wave functions (obtained as explained above) and a 151 × 151 × 151 integration grid. IQA and QTAIM calculations were performed with AIMALL,^106^ using the program’s default integration algorithms.

## Supplementary Material





## Notes

Cartesian coordinates (XYZ, comment line: name, method, electronic energy, Gibbs energy correction, imaginary frequency in transition states) (XYZ)
Results obtained by using different DFT exchange–correlation functionals, complete energy, BET, and IQA results for the whole set of systems under consideration; QTAIM charges; and activation and reaction energies with the ^t^Bu-based original system (PDF)
