# Supplementary material for: Metal–Ligand Cooperation in N–H Activation: Bridging Electron-Pushing Formalism and Energy Descriptors
Source: Inorg Chem. 2025 Oct 22;64(43):21452–64. doi: 10.1021/acs.inorgchem.5c03268 (PMC12587394; doi:10.1021/acs.inorgchem.5c03268)
Supplement: Supplementary file 2 [file ic5c03268_si_002.pdf]

Supporting Information for

Metal–Ligand Cooperation in N–H Activation: Bridging Electron-Pushing Formalism and Energy Descriptors

Daniel Barrena-Espés,[a] Victor Polo,[b] Jorge Echeverría,[c] Ángel Martín Pendás,[a] Julen Munárriz[b,*]

[a] Departamento de Química Física y Analítica, Universidad de Oviedo, Oviedo 33006, Spain.

[b] Departamento de Química Física and Instituto de Biocomputación y Física de Sistemas Complejos (BIFI), Universidad de Zaragoza, Zaragoza 50009, Spain. E-mail: julen@unizar.es.

[c] Departamento de Química Inorgánica and Instituto de Síntesis Química y Catálisis Homogénea (ISQCH), Universidad de Zaragoza, Pedro Cerbuna 12, Zaragoza 50009, Spain.

Comparison between different density functional approximations

Table S1. Electronic energy (in kcal·mol^{-1}) relative to **1-R**.

Density functional approximation	**1**	**1-R**	**1-TS**	**1-P**
B3LYP-D3BJ	21.4	0.0	**21.9**	**15.4**
BP86-D3BJ	23.2	0.0	**18.0**	**14.8**
M06	23.7	0.0	**22.3**	**14.8**
M06-L	23.4	0.0	**21.8**	**14.3**
M06-2X	23.2	0.0	**24.3**	**17.6**

Table S2. Gibbs energy (in kcal·mol^{-1}) relative to **1-R**.

Density functional approximation	**1**	**1-R**	**1-TS**	**1-P**
B3LYP-D3BJ	7.9	0.0	**19.4**	**14.4**
BP86-D3BJ	9.8	0.0	**15.5**	**13.7**
M06	10.3	0.0	**20.0**	**13.2**
M06-L	10.4	0.0	**19.3**	**13.2**
M06-2X	10.3	0.0	**21.9**	**17.0**

Summary of the BET results for the reaction of 1 with ammonia

Table S3. Results obtained for the BET analysis of dehydrogenation of ammonia for the three different structural stable domains. Each row shows: intrinsic reaction coordinate (amu$^{1/2}$·bohr), relative energy to **1-R** (kcal·mol^{-1}), distances of the main bonds (Å) and the ELF basin population (in e^{-}). Note that ECPs are employed in the case of Ru atoms, so that only 16 electrons are explicitly considered.

	SSD-I		Δ (I)	SSD-II		Δ (II)	SSD-III		Δ (III)
IRC	–8.88	–0.58	8.30	–0.54	0.04	0.58	0.08	6.20	6.12
E_{rel}	0.0	13.8	13.8	14.7	21.9	7.2	21.8	15.5	-6.3
d(H1,N1)	1.020	1.169	0.149	1.189	1.487	0.298	1.509	1.445	–0.064
d(H1,C1)	2.550	1.603	–0.947	1.582	1.295	–0.287	1.276	1.096	–0.180
d(C1,C2)	1.397	1.442	0.045	1.443	1.457	0.014	1.458	1.497	0.039
d(C3,C4)	1.365	1.376	0.012	1.377	1.381	0.004	1.381	1.385	0.004
d(C2,C3)	1.430	1.410	–0.020	1.409	1.401	–0.008	1.400	1.391	–0.010
d(N2,C2)	1.386	1.367	–0.020	1.366	1.360	–0.006	1.360	1.350	–0.009
d(N2,C6)	1.351	1.345	–0.006	1.345	1.346	0.001	1.346	1.350	0.004
d(C5,C6)	1.378	1.384	0.006	1.385	1.386	0.002	1.386	1.389	0.002
d(C4,C5)	1.409	1.397	–0.012	1.397	1.394	–0.003	1.393	1.387	–0.007
V(H1,N1)	1.99	2.09	0.10	-	-	-	-	-	-
V(H1)	-	-	-	0.64	0.70	0.06	-	-	-
V(H1,C1)	-	-	-	-	-	-	1.87	1.96	0.09
V(C1)	0.71	1.28	0.57	1.29	1.16	–0.13	-	-	-
V(C1,C2)	2.77	2.38	–0.39	2.37	2.22	–0.15	2.21	2.10	–0.11
V(C1,P1)	2.29	2.03	–0.26	2.03	1.98	–0.05	1.98	1.92	–0.06
V(N1,Ru)	1.93	1.89	–0.04	3.35	3.66	0.31	3.67	3.75	0.08

V(N2,Ru)	3.04	2.82	–0.22	2.81	2.72	–0.09	2.72	2.66	–0.06
V(C3,C4)	3.11	2.96	–0.15	2.96	2.86	–0.10	2.85	2.76	–0.09
V(C2,C3)	2.52	2.64	0.12	2.65	2.76	0.11	2.77	2.87	0.10
V(N2,C2)	2.15	2.27	0.12	2.28	2.35	0.07	2.35	2.43	0.08
V(N2,C6)	2.31	2.41	0.10	2.41	2.44	0.03	2.44	2.42	–0.02
V(C5,C6)	3.14	3.02	–0.12	3.01	2.96	–0.05	2.96	2.91	–0.05
V(C4,C5)	2.56	2.66	0.10	2.66	2.71	0.05	2.71	2.74	0.03

IQA results for the reaction of 1 with ammonia

Table S4. E_{int}, V_{xc} and V_{cl} (in kcal·mol^{-1}) for selected interactions in the reaction of **1-R** with ammonia.

	E_{int} (kcal·mol^{-1})			V_{xc} (kcal·mol^{-1})			V_{cl} (kcal·mol^{-1})		
	**1-R**	**1-TS**	**1-P**	**1-R**	**1-TS**	**1-P**	**1-R**	**1-TS**	**1-P**
N1–H1	–254.2	–142.75	–24.04	–152.3	–55.4	–6.7	–101.9	–87.3	–17.4
C1–H1	–50.5	–156.2	–162.9	–4.3	–89.7	–165.8	–46.3	–66.5	2.9
Ru–N1	–170.3	–202.0	–213.7	–60.4	–76.0	–86.8	–110.0	–125.9	–127.0
P1–C1	–629.8	–527.7	–436.7	–144.9	–133.3	–125.0	–484.8	–394.3	–311.7
C1–C2	–282.3	–248.6	–217.1	–261.5	–218.2	–196.9	–20.8	–30.4	–20.3
C2–C3	–202.8	–214.5	–221.0	–227.8	–244.9	–253.5	25.0	30.4	32.5
C3–C4	–249.2	–237.5	–232.6	–280.6	–266.5	–260.9	31.3	29.0	28.3
C4–C5	–218.1	–227.8	–213.7	–243.5	–255.0	–259.9	25.4	27.2	28.2
C5–C6	–231.8	–224.3	–222.3	–264.0	–256.9	–524.8	32.2	32.6	32.6
N2–C2	–370.6	–415.2	–431.7	–215.7	–225.5	–232.2	–154.8	–189.7	–199.4
N2–C6	–424.0	–437.9	–431.0	–233.3	–234.0	–233.7	–190.7	–203.9	–197.2
Ru–N2	–216.8	–215.9	–212.4	–79.0	–74.4	–73.8	–137.9	–141.5	–138.6
Ru–P1	28.2	32.9	29.7	–117.4	–115.9	–120.2	145.6	148.8	149.8
Ru–P2	5.0	9.8	11.9	–127.2	–129.6	–129.9	132.2	139.4	141.8
Ru–H2	–145.2	–145.8	–144.4	–132.0	–125.8	–120.9	–13.1	–20.0	–23.4
Ru–C7	–127.2	–121.0	–118.9	–215.2	–214.7	–214.3	88.0	93.8	95.3

Table S5. QTAIM charges (in |e$^-$|) for relevant atoms in the reaction of **1-R** with ammonia.

	**1-R**	**1-TS**	**1-P**
H1	0.39	0.34	0.11
N1	–1.02	–1.11	–1.12
C1	–0.86	–0.75	–0.52
Ru	0.67	0.70	0.71
P1	1.82	1.75	1.73
C2	0.38	0.44	0.47
C3	–0.05	–0.03	–0.02
C4	–0.03	–0.02	–0.02
C5	–0.06	–0.04	–0.03
C6	0.45	0.47	0.47
N2	–1.15	–1.15	–1.13

Complete set of amines

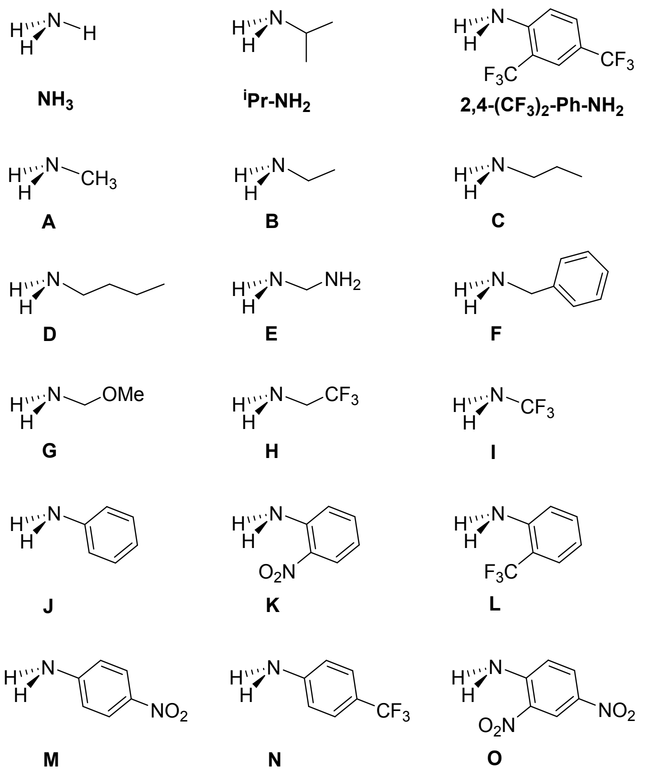

Chart S1. Complete set of amines considered for the reaction with **1**.

Table S6. Reaction and activation electronic and Gibbs energies (relative to **1-R'**) for the complete set of amines. Amine names are indicated in Chart S1 and are classified as electron withdrawing groups (EWG) or electron donor groups (EDG).

	ΔE (kcal·mol^{-1})				ΔG (kcal·mol^{-1})			
Amine	**1**	**1-R'**	**1-TS'**	**1-P'**	**1**	**1-R'**	**1-TS'**	**1-P'**
**NH_3** (EDG)	21.4	0.0	**21.9**	**15.4**	7.9	0.0	**19.4**	**14.4**
**^{i}Pr-NH_2** (EDG)	25.5	0.0	**22.6**	**16.9**	9.9	0.0	**19.7**	**15.8**
**2,4-$(CF_3)_2$-Ph-NH_2** (EWG)	17.6	0.0	**8.8**	**-12.0**	2.2	0.0	**7.1**	**-12.1**
**A** (EDG)	24.6	0.0	**23.6**	**17.3**	9.6	0.0	**20.6**	**16.0**
**B** (EDG)	25.1	0.0	**23.6**	**16.9**	10.3	0.0	**20.7**	**15.8**
**C** (EDG)	25.3	0.0	**23.7**	**17.0**	10.5	0.0	**20.6**	**15.9**
**D** (EDG)	25.5	0.0	**23.7**	**17.1**	10.5	0.0	**20.8**	**15.8**
**E** (EDG)	25.3	0.0	**22.9**	**15.4**	10.8	0.0	**20.4**	**14.7**
**F** (EDG)	25.9	0.0	**22.5**	**15.2**	11.2	0.0	**19.7**	**14.3**
**G** (EDG)	27.5	0.0	**21.8**	**12.1**	11.7	0.0	**18.7**	**11.0**
**H** (EDG)	22.5	0.0	**18.7**	**9.7**	7.8	0.0	**16.1**	**8.8**
**I** (EWG)	18.0	0.0	**9.8**	**-7.1**	2.9	0.0	**7.1**	**-7.6**
**J** (EWG)	21.4	0.0	**15.9**	**2.6**	5.9	0.0	**13.3**	**1.6**
**K** (EWG)	18.6	0.0	**10.4**	**-11.2**	2.1	0.0	**8.1**	**-12.8**
**L** (EWG)	18.9	0.0	**11.2**	**-6.6**	3.7	0.0	**9.5**	**-6.9**
**M** (EWG)	19.0	0.0	**11.5**	**-7.5**	3.6	0.0	**9.1**	**-8.0**
**N** (EWG)	24.5	0.0	**17.9**	**1.8**	7.5	0.0	**13.9**	**-0.7**
**O** (EWG)	19.9	0.0	**10.2**	**-15.8**	4.3	0.0	**8.1**	**-15.9**

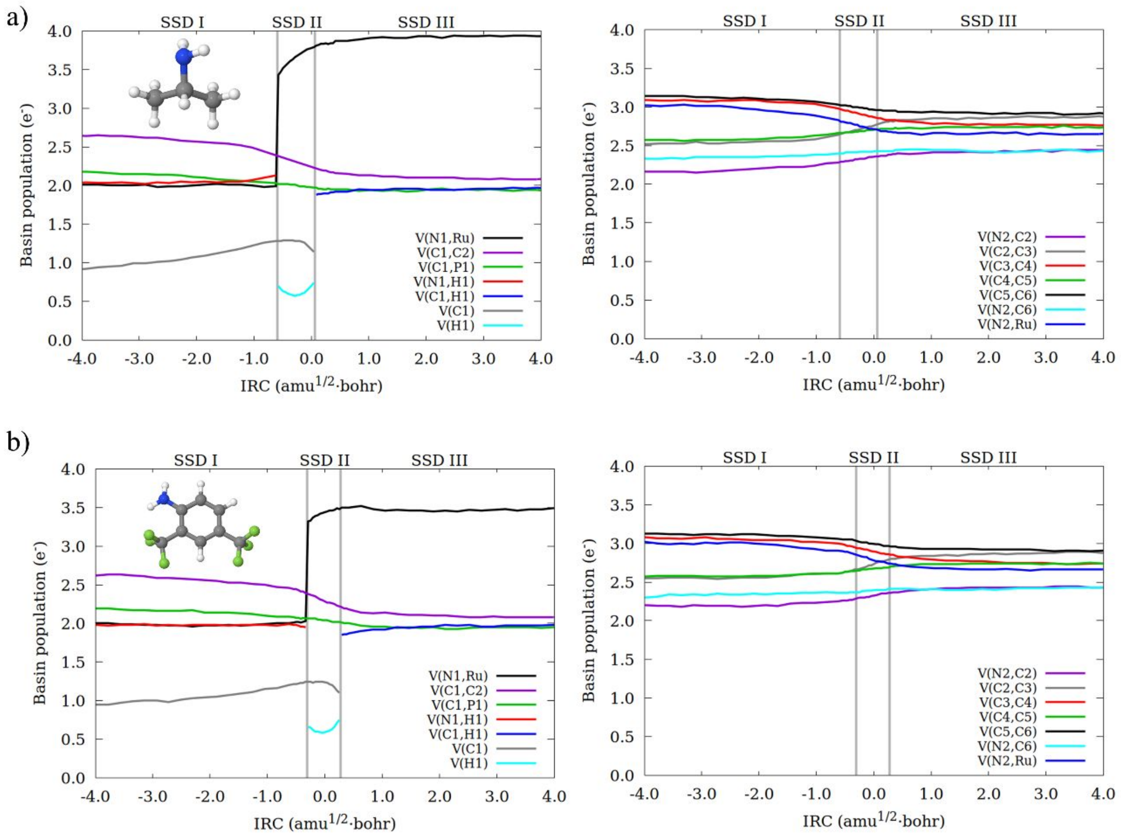

Figure S1. Integrated electron density (in electrons) for some ELF basins along the IRC path for the reaction of **1-R** with a) isopropylamine, b) 2,4-bis(trifluoromethyl)aniline. Bifurcation points separating the SSDs are indicated by vertical lines.

Table S7. Results obtained for the BET analysis of dehydrogenation of isopropylamine for the three different structural stable domains. Each row shows: intrinsic reaction coordinate ($amu^{1/2}\cdot bohr$), relative energy to **1-R'** ($kcal\cdot mol^{-1}$), distances of the main bonds (Å) and the ELF basin population (in e^{-}).

	SSD-I		Δ (I)	SSD-II		Δ (II)	SSD-III		Δ (III)
IRC	–4.55	–0.62	3.93	–0.58	0.04	0.62	0.08	5.17	5.09
E_{rel}	0.3	13.44	13.1	14.35	22.6	8.2	22.5	17.3	–5.2
d(H1,N1)	1.025	1.167	0.142	1.189	1.510	0.321	1.532	2.414	0.882
d(H1,C1)	2.285	1.612	–0.673	1.593	1.284	–0.309	1.266	1.096	–0.170
d(C1,C2)	1.398	1.441	0.043	1.442	1.457	0.015	1.458	1.496	0.038
d(C3,C4)	1.366	1.376	0.010	1.376	1.381	0.005	1.381	1.385	0.004
d(C2,C3)	1.427	1.410	–0.017	1.409	1.401	–0.008	1.400	1.391	–0.009
d(N2,C2)	1.386	1.367	–0.019	1.367	1.361	–0.006	1.360	1.350	–0.010
d(N2,C6)	1.350	1.345	–0.005	1.345	1.346	0.001	1.346	1.350	0.004
d(C5,C6)	1.379	1.384	0.005	1.384	1.386	0.002	1.386	1.388	0.002
d(C4,C5)	1.408	1.397	–0.011	1.397	1.394	–0.003	1.393	1.387	–0.006
V(H1,N1)	2.03	2.13	0.10	-	-	-	-	-	-
V(H1)	-	-	-	0.70	0.74	0.04	-	-	-
V(H1,C1)	-	-	-	-	-	-	1.88	1.97	0.09

V(C1)	0.90	1.28	0.38	1.28	1.14	–0.14	-	-	-
V(C1,C2)	2.66	2.39	–0.27	2.38	2.23	–0.15	2.22	2.08	–0.14
V(C1,P1)	2.19	2.03	–0.16	2.02	1.97	–0.05	1.97	1.94	–0.03
V(N1,Ru)	2.00	1.99	–0.01	3.43	3.79	0.36	3.81	3.95	0.14
V(N2,Ru)	3.02	2.83	–0.19	2.81	2.71	–0.10	2.70	2.64	–0.06
V(C3,C4)	3.09	2.98	–0.11	2.97	2.86	–0.11	2.85	2.77	–0.08
V(C2,C3)	2.51	2.64	0.13	2.65	2.77	0.12	2.78	2.87	0.09
V(N2,C2)	2.17	2.28	0.11	2.29	2.36	0.07	2.36	2.44	0.08
V(N2,C6)	2.34	2.40	0.06	2.40	2.43	0.03	2.43	2.44	0.01
V(C5,C6)	3.14	3.03	–0.11	3.02	2.96	–0.06	2.96	2.92	–0.04
V(C4,C5)	2.56	2.66	0.10	2.66	2.71	0.05	2.71	2.74	0.03

Table S8. Results obtained for the BET analysis of dehydrogenation of 2,4-bis(trifluoromethyl)aniline for the three different structural stable domains. Each row shows: intrinsic reaction coordinate (amu$^{1/2}$·bohr), relative energy to **1-R'** (kcal·mol^{-1}), distances of the main bonds (Å) and the ELF basin population (in e^{-}).

	SSD-I		Δ (I)	SSD-II		Δ (II)	SSD-III		Δ (III)
IRC	–9.86	–0.34	9.52	–0.29	0.25	0.5447	0.29	11.32	11.03
E_{rel}	0.3	6.8	6.5	7.2	6.9	–0.2	6.3	–11.7	–18.0
d(H1,N1)	1.031	1.152	0.121	1.172	1.450	0.278	1.471	2.742	1.271
d(H1,C1)	2.289	1.615	–0.674	1.594	1.320	–0.274	1.299	1.094	–0.205
d(C1,C2)	1.402	1.437	0.035	1.439	1.457	0.018	1.454	1.501	0.047
d(C3,C4)	1.365	1.375	0.010	1.375	1.379	0.004	1.380	1.385	0.005
d(C2,C3)	1.427	1.410	–0.017	1.410	1.401	–0.009	1.401	1.389	–0.012
d(N2,C2)	1.384	1.369	–0.015	1.369	1.363	–0.006	1.362	1.351	–0.011
d(N2,C6)	1.353	1.348	–0.005	1.348	1.349	0.001	1.349	1.351	0.002
d(C5,C6)	1.377	1.382	0.005	1.382	1.384	0.002	1.384	1.388	0.004
d(C4,C5)	1.408	1.399	–0.009	1.398	1.395	–0.003	1.394	1.386	–0.008
V(H1,N1)	1.97	1.95	–0.02	-	-	-	-	-	-
V(H1)	-	-	-	0.66	0.75	0.09	-	-	-
V(H1,C1)	-	-	-	-	-	-	1.85	1.97	0.12
V(C1)	0.80	1.24	0.44	1.24	1.10	–0.14	-	-	-
V(C1,C2)	2.71	2.39	–0.32	2.38	2.22	–0.16	2.21	2.08	–0.13
V(C1,P1)	2.27	2.06	–0.21	2.06	2.02	–0.04	2.01	1.96	–0.05
V(N1,Ru)	1.99	2.03	0.04	3.32	3.48	0.16	3.50	3.47	–0.03
V(N2,Ru)	3.04	2.86	–0.18	2.85	2.74	–0.11	2.74	2.65	–0.09
V(C3,C4)	3.09	2.95	–0.14	2.94	2.86	–0.08	2.85	2.77	–0.08
V(C2,C3)	2.53	2.66	0.13	2.66	2.79	0.13	2.80	2.88	0.08
V(N2,C2)	2.15	2.28	0.13	2.29	2.36	0.07	2.36	2.44	0.08
V(N2,C6)	2.32	2.37	0.05	2.37	2.41	0.04	2.41	2.42	0.01
V(C5,C6)	3.14	3.04	–0.10	3.03	2.97	–0.06	2.96	2.89	–0.07
V(C4,C5)	2.57	2.64	0.07	2.65	2.69	0.04	2.7	2.74	0.04

Table S9. E_{int}, V_{xc} and V_{cl} (in kcal·mol^{-1}) for selected interactions in the reaction of **1-R’** with isopropylamine.

	E_{int} (kcal·mol^{-1})			V_{xc} (kcal·mol^{-1})			V_{cl} (kcal·mol^{-1})		
	**1-R’**	**1-TS’**	**1-P’**	**1-R’**	**1-TS’**	**1-P’**	**1-R’**	**1-TS’**	**1-P’**
N1–H1	–246.8	–134.9	–23.7	–156.4	–53.6	–7.1	–90.4	–81.3	–16.6
C1–H1	–42.6	–155.1	–162.4	–2.8	–92.7	–165.5	–39.8	–62.5	3.1
Ru–N1	–170.6	–199.8	–214.2	–60.8	–76.1	–86.4	–109.8	–123.7	–127.8
P1–C1	–628.1	–524.4	–437.4	–145.0	–132.9	–125.1	–483.1	–391.5	–312.3
C1–C2	–283.5	–247.6	–217.4	–263.5	–217.9	–197.1	–20.0	–29.7	–20.3
C2–C3	–202.3	–214.6	–220.9	–227.2	–245.1	–253.5	24.9	30.4	32.5
C3–C4	–249.8	–237.4	–232.6	–281.2	–266.5	–260.9	31.4	29.0	28.3
C4–C5	–217.6	–227.7	–231.7	–242.9	–254.9	–259.9	25.3	27.2	28.2
C5–C6	–232.3	–224.3	–222.2	–264.3	–256.9	–254.8	32.0	32.7	32.6
N2–C2	–369.0	–414.1	–431.7	–214.7	–225.5	–232.1	–154.3	–188.8	–199.5
N2–C6	–422.5	–437.8	–431.2	–233.3	–233.8	–233.6	–189.1	–204.0	–197.6
Ru–N2	–218.0	–216.4	–212.5	–78.7	–74.8	–73.6	–139.3	–141.7	–138.9
Ru–P1	28.6	32.5	30.3	–117.5	–115.8	–119.9	146.1	148.2	150.2
Ru–P2	6.2	9.7	12.7	–126.7	–129.2	–128.6	132.9	138.9	141.3
Ru–H2	–144.8	–145.3	–144.0	–131.0	–125.0	–120.4	–13.8	–20.3	–23.6
Ru–C7	–127.2	–120.9	–117.8	–215.5	–214.1	–213.6	88.3	93.2	95.8

Table S10. E_{int}, V_{xc} and V_{cl} (in kcal·mol^{-1}) for selected interactions in the reaction of **1-R’** with 2,4-bis(trifluoromethyl)aniline.

	E_{int} (kcal·mol^{-1})			V_{xc} (kcal·mol^{-1})			V_{cl} (kcal·mol^{-1})		
	**1-R’**	**1-TS’**	**1-P’**	**1-R’**	**1-TS’**	**1-P’**	**1-R’**	**1-TS’**	**1-P’**
N1–H1	–259.2	–173.0	–13.7	–143.9	–71.7	–2.1	–115.3	–101.3	–11.6
C1–H1	–59.9	–136.5	–161.3	–6.9	–69.0	–168.9	–53.0	–67.4	7.6
Ru–N1	–155.2	–179.4	–215.5	–48.2	–59.5	–77.9	–107.0	–120.0	–137.6
P1–C1	–630.5	–549.1	–435.3	–145.2	–135.4	–125.2	–485.3	–413.7	–310.0
C1–C2	–280.7	–253.7	–215.5	–258.5	–223.9	–195.5	–22.3	–29.8	–20.0
C2–C3	–203.5	–212.6	–220.5	–229.1	–242.7	–253.8	25.7	30.1	33.3
C3–C4	–248.5	–239.0	–232.4	–279.7	–268.4	–260.9	31.2	29.4	28.5
C4–C5	–218.4	–226.1	–231.4	–243.9	–253.0	–259.7	25.5	27.0	28.2
C5–C6	–231.9	–225.7	–222.1	–264.5	–258.8	–255.2	32.7	33.2	33.1
N2–C2	–373.2	–407.8	–432.4	–216.1	–223.8	–232.6	–157.1	–184.0	–199.8
N2–C6	–421.1	–433.8	–430.0	–232.0	–232.4	–232.7	–189.2	–201.4	–197.3
Ru–N2	–216.1	–215.8	–214.4	–79.7	–76.3	–73.5	–136.4	–139.6	–140.9
Ru–P1	23.2	28.5	25.3	–116.4	–113.9	–121.5	139.6	142.4	146.8
Ru–P2	1.3	6.4	19.9	–127.1	–128.4	–125.7	128.4	134.8	145.6
Ru–H2	–143.2	–144.8	–146.0	–134.2	–131.0	–126.3	–9.0	–13.9	–19.7
Ru–C7	–129.7	–122.1	–114.9	–215.1	–212.6	–211.7	85.4	90.5	96.8

Table S11. QTAIM charges (in |e⁻|) for relevant atoms in the reaction of 1-**R'** with isopropylamine.

	**1-R'**	**1-TS'**	**1-P'**
H1	0.36	0.33	0.11
N1	–0.98	–1.05	–1.08
C1	–0.85	–0.74	–0.52
Ru	0.67	0.70	0.71
P1	1.82	1.74	1.73
C2	0.38	0.44	0.47
C3	–0.05	–0.03	–0.02
C4	–0.03	–0.02	–0.02
C5	–0.06	–0.04	–0.02
C6	0.45	0.47	0.47
N2	–1.15	1.15	–1.13

Table S12. QTAIM charges (in |e⁻|) for relevant atoms in the reaction of 1-**R'** with 2,4-bis(trifluoromethyl)aniline.

	**1-R'**	**1-TS'**	**1-P'**
H1	0.41	0.36	0.09
N1	–1.05	–1.10	–1.16
C1	–0.87	–0.78	–0.51
Ru	0.65	0.67	0.72
P1	1.81	1.75	1.72
C2	0.38	0.43	0.47
C3	–0.04	–0.03	–0.01
C4	–0.03	–0.02	–0.01
C5	–0.05	–0.03	–0.02
C6	0.45	0.46	0.47
N2	–1.15	–1.16	–1.14

Table S13. E_{int}, V_{xc} and V_{cl} (in kcal·mol^{-1}) for interactions between groups (defined according to Scheme 4) in the reaction of **1-R'** with ammonia.

Interaction	V_{xc} (kcal·mol^{-1})			V_{cl} (kcal·mol^{-1})			E_{int} (kcal·mol^{-1})		
	R'	TS'	P'	R'	TS'	P'	R'	TS'	P'
NH– [Ru]	–65.5	–82.9	–95.5	–44.7	–55.2	–54.6	–110.2	–138.1	–150.1
NH–H1	–153.4	–55.9	–6.8	–64.5	–67.7	–13.8	–217.9	–123.5	–20.5
NH–R	–163.2	–170.4	–174.2	–54.0	–47.1	–41.4	–217.3	–217.5	–215.6
NH–PNP	–21.3	–36.1	–34.2	23.5	7.9	–31.5	2.3	–28.2	–65.7
[Ru]–H1	–0.9	–1.1	–0.3	20.8	18.7	4.5	19.9	17.7	4.2
[Ru]–R	–1.0	–1.4	–2.1	14.6	14.6	13.9	13.6	13.3	11.8
[Ru]–PNP	–390.0	–387.7	–393.3	–14.4	–5.7	8.5	–404.4	–393.3	–384.8
R–H1	–1.1	–0.5	–0.1	37.9	19.8	3.5	36.8	19.4	3.4
PNP–H1	–7.4	–99.3	–178.2	–17.7	–8.7	30.5	–25.1	–107.9	–147.7
PNP–R	–1.0	–1.3	–0.8	–9.8	–1.5	9.6	–10.8	–2.8	8.8

Table S14. E_{int}, V_{xc} and V_{cl} (in kcal·mol^{-1}) for interactions between groups (defined according to Scheme 4) in the reaction of **1-R'** with isopropylamine.

Interaction	V_{xc} (kcal·mol^{-1})			V_{cl} (kcal·mol^{-1})			E_{int} (kcal·mol^{-1})		
	R'	TS'	P'	R'	TS'	P'	R'	TS'	P'
NH–[Ru]	–65.3	–81.9	–93.3	–42.8	–55.0	–57.1	–108.1	–137.0	–150.4
NH–H1	–157.6	–54.1	–7.2	–55.1	–63.0	–13.1	–212.7	–117.1	–20.3
NH–R	–209.3	–225.2	–232.8	–84.8	–77.1	–70.1	–294.1	–302.3	–302.8
NH–PNP	–20.9	–34.2	–30.1	21.1	7.2	–29.6	0.2	–27.0	–59.6
[Ru]–H1	–0.9	–1.1	–0.3	18.4	17.8	4.5	17.5	16.7	4.2
[Ru]–R	–9.9	–10.3	–12.3	15.4	16.3	16.3	5.6	6.0	3.9
[Ru]–PNP	–389.4	–387.3	–391.1	–15.0	–5.9	7.9	–404.5	–393.2	–383.2
R–H1	–3.6	–1.6	–0.4	37.6	19.9	3.5	34.0	18.3	3.1
PNP–H1	–5.4	–102.2	–177.8	–14.7	–7.0	30.2	–20.1	–109.2	–147.6
PNP–R	–11.2	–12.2	–14.1	–10.1	–1.7	9.0	–21.4	–13.9	–5.1

Table S15. E_{int}, V_{xc} and V_{cl} (in kcal·mol^{-1}) for interactions between groups (defined according to Scheme 4) in the reaction of **1-R'** with 2,4-bis(trifluoromethyl)aniline.

Interaction	V_{xc} (kcal·mol^{-1})			V_{cl} (kcal·mol^{-1})			E_{int} (kcal·mol^{-1})		
	R'	TS'	P'	R'	TS'	P'	R'	TS'	P'
NH–[Ru]	–51.4	–63.5	–83.2	–41.1	–52.0	–62.4	–92.5	–115.6	–145.6
NH–H1	–144.8	–72.4	–2.3	–73.4	–73.4	–8.2	–218.2	–145.7	–10.4
NH–R	–234.9	–257.4	–288.7	–102.1	–107.5	–146.8	–337.0	–364.9	–435.5
NH–PNP	–20.5	–30.8	–27.5	21.2	9.6	–35.0	0.7	–21.3	–62.5
[Ru]–H1	–0.9	–1.2	–0.2	20.9	20.8	3.6	20.0	19.6	3.4
[Ru]–R	–11.2	–11.3	–12.1	14.9	17.7	22.5	3.7	6.4	10.4
[Ru]–PNP	–389.4	–384.2	–386.9	–15.7	–8.7	9.9	–405.0	–393.0	–377.0
R–H1	–2.6	–1.9	–2.9	45.4	29.7	2.2	42.8	27.7	-0.8
PNP–H1	–11.0	–78.0	–181.3	–18.2	–11.9	30.6	–29.2	–89.9	–150.8
PNP–R	–13.0	–15.6	–12.4	–10.1	–4.1	9.0	–23.1	–19.7	–3.4

Structural modifications of 1

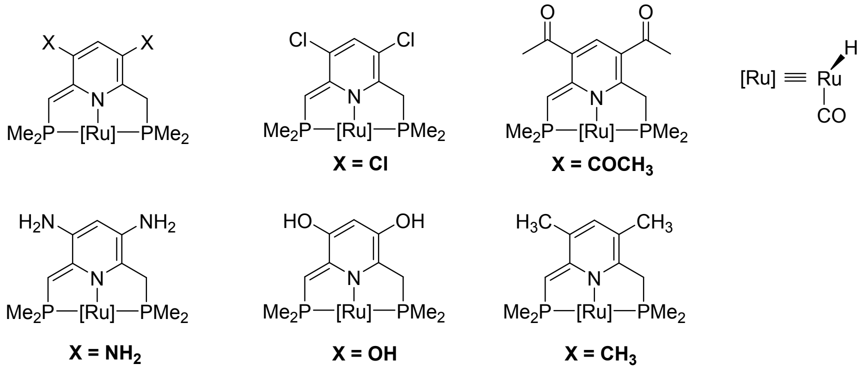

Chart S2. Modifications to **1** considered in this work. Note that systems with X = Cl and $COCH_3$ are considered as electron withdrawing, while X = NH_2, OH and CH_3 are considered as electron donor.

Table S16. Electronic energies for the reaction of the modified systems with ammonia relative to **1-R[X]** (in kcal·mol^{-1}).

X =	**1[X]**	**1-R[X]**	**1-TS[X]**	**1-P[X]**
Cl	21.9	0.0	24.1	18.8
$COCH_3$	24.1	0.0	25.7	19.4
NH_2	21.8	0.0	19.0	10.0
OH	21.3	0.0	20.2	11.8
CH_3	21.5	0.0	21.2	14.5

Table S17. Gibbs energies for the reaction of the modified systems with ammonia relative to **1-R[X]** (in kcal·mol^{-1}).

X =	**1[X]**	**1-R[X]**	**1-TS[X]**	**1-P[X]**
Cl	8.4	0.0	21.5	17.2
$COCH_3$	10.0	0.0	21.7	17.2
NH_2	8.1	0.0	16.6	9.3
OH	7.8	0.0	17.9	11.1
CH_3	7.9	0.0	18.6	13.3

Table S18. Results obtained for the BET analysis for the reaction of ammonia with **1[NH$_2$]**. Each row shows: intrinsic reaction coordinate (amu$^{1/2}$·bohr), relative energy to **1[NH$_2$]** (kcal·mol^{-1}), distances of the main bonds (Å) and the ELF basin population (in e^-).

	SSD-I		Δ (I)	SSD-II		Δ (II)	SSD-III		Δ (III)
IRC	–6.16	–0.53	5.63	–0.49	0.12	0.62	0.16	4.80	4.64
E_{rel}	0.3	12.0	11.7	12.8	18.7	5.9	18.5	10.3	–8.2
d(N1,H1)	1.022	1.159	0.137	1.179	1.496	0.317	1.518	2.506	0.988
d(C1,H1)	2.409	1.606	–0.803	1.585	1.279	–0.306	1.261	1.095	–0.166
d(C1,C2)	1.415	1.457	0.042	1.458	1.470	0.012	1.471	1.500	0.029
d(C3,C4)	1.372	1.382	0.010	1.382	1.386	0.004	1.386	1.389	0.003

d(C2,C3)	1.437	1.420	–0.017	1.420	1.412	–0.008	1.411	1.404	–0.007
d(N2,C2)	1.364	1.351	–0.013	1.351	1.347	–0.004	1.346	1.344	–0.002
d(N2,C6)	1.364	1.352	–0.012	1.352	1.350	–0.002	1.350	1.347	–0.003
d(C5,C6)	1.378	1.388	0.010	1.389	1.392	0.003	1.393	1.401	0.008
d(C4,C5)	1.408	1.399	–0.009	1.399	1.396	–0.003	1.396	1.391	–0.005
V(N1,H1)	2.01	2.09	0.08	-	-	-	-	-	-
V(H1)	-	-	-	0.64	0.71	0.07	-	-	-
V(C1,H1)	-	-	-	-	-	-	1.92	1.99	0.07
V(C1)	1.05	1.39	0.34	1.39	1.21	–0.18	-	-	-
V(C1,C2)	2.57	2.34	–0.23	2.33	2.19	–0.14	2.18	2.07	–0.11
V(C1,P1)	2.19	2.02	–0.17	2.02	1.98	–0.04	1.97	1.94	–0.03
V(N1,Ru)	1.93	1.89	–0.04	3.35	3.67	0.32	3.68	3.79	0.11
V(C1,H2)	2.06	2.05	–0.01	2.06	2.05	–0.01	2.05	2.02	–0.03
V(Ru,P1)	2.06	2.09	0.03	2.09	2.11	0.02	2.11	2.14	0.03
V(Ru,P2)	2.23	2.25	0.02	2.26	2.25	–0.01	2.25	2.26	0.01
V(Ru,C7)	3.26	3.23	–0.03	3.23	3.22	–0.01	3.21	3.24	0.03
V(Ru,H2)	1.75	1.77	0.02	1.77	1.78	0.01	1.78	1.81	0.03
C(Ru)	13.27	13.26	–0.01	13.26	13.24	–0.02	13.24	13.21	–0.03
V(N2,Ru)	2.95	2.77	–0.18	2.76	2.65	–0.11	2.64	2.59	–0.05
V(C3,C4)	3.14	3.02	–0.12	3.02	2.93	–0.09	2.93	2.86	–0.07
V(C2,C3)	2.58	2.71	0.13	2.72	2.84	0.12	2.84	2.98	0.14
V(N2,C2)	2.30	2.41	0.11	2.42	2.48	0.06	2.48	2.48	0.00
V(N2,C6)	2.23	2.33	0.10	2.34	2.39	0.05	2.39	2.45	0.06
V(C5,C6)	3.33	3.19	–0.14	3.19	3.12	–0.07	3.12	3.05	–0.07
V(C4,C5)	2.66	2.74	0.08	2.75	2.80	0.05	2.80	2.83	0.03

Table S19. Results obtained for the BET analysis for the reaction of ammonia with **1[Cl]**. Each row shows: intrinsic reaction coordinate (amu$^{1/2}$·bohr), relative energy to **1[Cl]** (kcal·mol^{-1}), distances of the main bonds (Å) and the ELF basin population (in e^{-}).

	SSD-I		Δ (I)	SSD-II		Δ (II)	SSD-III		Δ (III)
IRC	–7.87	–0.62	7.25	–0.58	0.00	0.58	0.04	3.73	3.69
E_{rel}	0.3	14.9	14.6	15.9	24.1	8.3	24.1	19.0	–5.1
d(N1,H1)	1.020	1.167	0.147	1.187	1.486	0.299	1.508	2.386	0.878
d(C1,H1)	2.561	1.607	–0.954	1.586	1.298	–0.288	1.280	1.101	–0.179
d(C1,C2)	1.387	1.436	0.049	1.437	1.452	0.015	1.453	1.492	0.039
d(C3,C4)	1.363	1.374	0.011	1.374	1.378	0.004	1.378	1.383	0.005
d(C2,C3)	1.436	1.415	–0.021	1.414	1.406	–0.008	1.406	1.395	–0.011
d(N2,C2)	1.389	1.367	–0.022	1.366	1.360	–0.006	1.360	1.350	–0.010
d(N2,C6)	1.351	1.345	–0.006	1.345	1.346	0.001	1.346	1.350	0.004
d(C5,C6)	1.380	1.388	0.008	1.388	1.390	0.002	1.390	1.393	0.003
d(C4,C5)	1.404	1.393	–0.011	1.393	1.390	–0.003	1.390	1.384	–0.006
V(N1,H1)	1.98	2.08	0.10	-	-	-	-	-	-
V(H1)	-	-	-	0.67	0.70	0.03	-	-	-
V(C1,H1)	-	-	-	-	-	-	1.85	1.95	0.10

V(C1)	0.63	1.25	0.62	1.25	1.15	–0.10	-	-	-
V(C1,C2)	2.88	2.44	–0.44	2.43	2.28	–0.15	2.27	2.14	–0.13
V(C1,P1)	2.26	2.00	–0.26	2.00	1.96	–0.04	1.95	1.92	–0.03
V(N1,Ru)	1.93	1.89	–0.04	3.32	3.66	0.34	3.68	3.78	0.10
V(C1,H2)	2.08	2.07	–0.01	2.07	2.07	0.00	2.06	2.00	–0.06
V(Ru,P1)	2.09	2.10	0.01	2.10	2.11	0.01	2.12	2.15	0.03
V(Ru,P2)	2.22	2.25	0.03	2.25	2.25	0.00	2.25	2.28	0.03
V(Ru,C7)	3.25	3.23	–0.02	3.24	3.23	–0.01	3.23	3.22	–0.01
V(Ru,H2)	1.74	1.76	0.02	1.76	1.78	0.02	1.78	1.80	0.02
C(Ru)	13.25	13.23	–0.02	13.23	13.21	–0.02	13.21	13.19	–0.02
V(N2,Ru)	3.06	2.85	–0.21	2.84	2.74	–0.10	2.73	2.64	–0.09
V(C3,C4)	3.25	3.10	–0.15	3.08	2.97	–0.11	2.96	2.86	–0.10
V(C2,C3)	2.56	2.71	0.15	2.73	2.85	0.12	2.86	2.98	0.12
V(N2,C2)	2.14	2.30	0.16	2.30	2.37	0.07	2.37	2.43	0.06
V(N2,C6)	2.32	2.40	0.08	2.41	2.43	0.02	2.44	2.42	–0.02
V(C5,C6)	3.33	3.17	–0.16	3.16	3.09	–0.07	3.08	3.03	–0.05
V(C4,C5)	2.65	2.76	0.11	2.76	2.81	0.05	2.81	2.85	0.04

Table S20. Results obtained for the BET analysis for the reaction of ammonia with **1[CH$_3$]**. Each row shows: intrinsic reaction coordinate (amu$^{1/2}$·bohr), relative energy to **1[CH$_3$]** (kcal·mol^{-1}), distances of the main bonds (Å) and the ELF basin population (in e^-).

	SSD-I		Δ (I)	SSD-II		Δ (II)	SSD-III		Δ (III)
IRC	–7.22	–0.58	6.64	–0.54	0.04	0.58	0.08	4.95	4.87
E_{rel}	0.3	13.2	12.9	14.0	21.2	7.2	21.1	14.7	–6.4
d(N1,H1)	1.022	1.162	0.140	1.182	1.479	0.297	1.501	2.431	0.930
d(C1,H1)	2.469	1.608	–0.861	1.587	1.299	–0.288	1.280	1.100	–0.180
d(C1,C2)	1.401	1.447	0.046	1.448	1.461	0.013	1.462	1.498	0.036
d(C3,C4)	1.370	1.380	0.010	1.380	1.383	0.003	1.384	1.388	0.004
d(C2,C3)	1.435	1.416	–0.019	1.415	1.408	–0.007	1.407	1.398	–0.009
d(N2,C2)	1.382	1.363	–0.019	1.363	1.357	–0.006	1.357	1.349	–0.008
d(N2,C6)	1.354	1.347	–0.007	1.347	1.347	0.000	1.347	1.349	0.002
d(C5,C6)	1.383	1.390	0.007	1.390	1.392	0.002	1.392	1.396	0.004
d(C4,C5)	1.409	1.399	–0.010	1.399	1.396	–0.003	1.396	1.389	–0.007
V(N1,H1)	1.98	2.09	0.11	-	-	-	-	-	-
V(H1)	-	-	-	0.65	0.69	0.04	-	-	-
V(C1,H1)	-	-	-	-	-	-	1.87	1.96	0.09
V(C1)	0.79	1.30	0.51	1.30	1.17	–0.13	-	-	-
V(C1,C2)	2.71	2.37	–0.34	2.36	2.23	–0.13	2.22	2.06	–0.16
V(C1,P1)	2.25	2.03	–0.22	2.02	1.97	–0.05	1.98	1.93	–0.05
V(N1,Ru)	1.92	1.89	–0.03	3.34	3.66	0.32	3.68	3.77	0.09
V(C1,H2)	2.09	2.06	–0.03	2.06	2.08	0.02	2.07	2.03	–0.04
V(Ru,P1)	2.08	2.10	0.02	2.10	2.12	0.02	2.12	2.15	0.03
V(Ru,P2)	2.24	2.25	0.01	2.25	2.25	0.00	2.25	2.25	0.00
V(Ru,C7)	3.24	3.23	–0.01	3.23	3.22	–0.01	3.22	3.20	–0.02

V(Ru,H2)	1.75	1.76	0.01	1.76	1.78	0.02	1.78	1.81	0.03
C(Ru)	13.26	13.25	–0.01	13.24	13.24	0.00	13.24	13.23	–0.01
V(N2,Ru)	3.01	2.80	–0.21	2.79	2.69	–0.10	2.68	2.63	–0.05
V(C3,C4)	3.13	2.98	–0.15	2.98	2.91	–0.07	2.91	2.81	–0.10
V(C2,C3)	2.55	2.68	0.13	2.69	2.79	0.10	2.79	2.92	0.13
V(N2,C2)	2.17	2.31	0.14	2.31	2.39	0.08	2.39	2.44	0.05
V(N2,C6)	2.32	2.39	0.07	2.39	2.43	0.04	2.42	2.44	0.02
V(C5,C6)	3.17	3.06	–0.11	3.06	3.00	–0.06	3.00	2.95	–0.05
V(C4,C5)	2.64	2.73	0.09	2.73	2.77	0.04	2.78	2.79	0.01

Table S21. Results obtained for the BET analysis for the reaction of ammonia with **1[OH]**. Each row shows: intrinsic reaction coordinate ($amu^{1/2}\cdot bohr$), relative energy to **1[OH]** ($kcal\cdot mol^{-1}$), distances of the main bonds (Å) and the ELF basin population (in e^{-}).

	SSD-I		Δ (I)	SSD-II		Δ (II)	SSD-III		Δ (III)
IRC	–7.62	–0.58	7.04	–0.54	0.08	0.62	0.12	4.74	4.61
E_{rel}	0.3	12.2	11.9	13.0	20.1	7.1	20.0	12.1	–7.9
d(N1,H1)	1.021	1.148	0.127	1.168	1.485	0.317	1.506	2.492	0.986
d(C1,H1)	2.476	1.623	–0.853	1.602	1.293	–0.309	1.274	1.089	–0.185
d(C1,C2)	1.401	1.446	0.045	1.447	1.461	0.014	1.462	1.497	0.035
d(C3,C4)	1.367	1.378	0.011	1.378	1.382	0.004	1.382	1.387	0.005
d(C2,C3)	1.434	1.415	–0.019	1.414	1.406	–0.008	1.406	1.396	–0.010
d(N2,C2)	1.371	1.355	–0.016	1.355	1.350	–0.005	1.350	1.345	–0.005
d(N2,C6)	1.362	1.350	–0.012	1.350	1.349	–0.001	1.349	1.347	–0.002
d(C5,C6)	1.372	1.382	0.010	1.382	1.386	0.004	1.386	1.393	0.007
d(C4,C5)	1.409	1.398	–0.011	1.398	1.395	–0.003	1.395	1.388	–0.007
V(N1,H1)	2.00	2.09	0.09	-	-	-	-	-	-
V(H1)	-	-	-	0.66	0.70	0.04	-	-	-
V(C1,H1)	-	-	-	-	-	-	1.88	1.97	0.09
V(C1)	0.80	1.31	0.51	1.31	1.17	–0.14	-	-	-
V(C1,C2)	2.70	2.39	–0.31	2.38	2.22	–0.16	2.22	2.07	–0.15
V(C1,P1)	2.27	2.03	–0.24	2.02	1.98	–0.04	1.97	1.94	–0.03
V(N1,Ru)	1.93	1.88	–0.05	3.32	3.68	0.36	3.69	3.79	0.10
V(C1,H2)	2.09	2.07	–0.02	2.07	2.07	0.00	2.06	2.03	–0.03
V(Ru,P1)	2.07	2.08	0.01	2.09	2.11	0.02	2.11	2.14	0.03
V(Ru,P2)	2.23	2.24	0.01	2.24	2.24	0.00	2.24	2.25	0.01
V(Ru,C7)	3.24	3.22	–0.02	3.22	3.22	0.00	3.21	3.21	0.00
V(Ru,H2)	1.75	1.77	0.02	1.76	1.78	0.02	1.78	1.81	0.03
C(Ru)	13.27	13.25	–0.02	13.24	13.23	–0.01	13.22	13.19	–0.03
V(N2,Ru)	3.02	2.79	–0.23	2.78	2.66	–0.12	2.67	2.61	–0.06
V(C3,C4)	3.25	3.11	–0.14	3.10	3.02	–0.08	3.02	2.92	–0.10
V(C2,C3)	2.60	2.74	0.14	2.75	2.87	0.12	2.88	2.99	0.11
V(N2,C2)	2.26	2.36	0.10	2.37	2.44	0.07	2.44	2.46	0.02
V(N2,C6)	2.23	2.33	0.10	2.34	2.39	0.05	2.39	2.46	0.07
V(C5,C6)	3.37	3.22	–0.15	3.22	3.13	–0.09	3.13	3.06	–0.07
V(C4,C5)	2.67	2.77	0.10	2.77	2.83	0.06	2.84	2.91	0.07

Table S22. Results obtained for the BET analysis for the reaction of ammonia with **1[COCH$_3$]**. Each row shows: intrinsic reaction coordinate (amu$^{1/2}$·bohr), relative energy to **1[COCH$_3$]** (kcal·mol^{-1}), distances of the main bonds (Å) and the ELF basin population (in e^{-}).

	SSD-I		Δ (I)	SSD-II		Δ (II)	SSD-III		Δ (III)
IRC	–14.48	–0.62	13.86	–0.58	0.04	0.52	0.08	10.34	10.42
E_{rel}	1.6	16.5	14.9	17.4	25.6	8.2	25.6	19.5	–6.1
d(N1,H1)	1.019	1.159	0.140	1.180	1.500	0.320	1.521	2.494	0.973
d(C1,H1)	2.622	1.604	–1.018	1.591	1.285	–0.306	1.267	1.095	–0.172
d(C1,C2)	1.389	1.440	0.051	1.441	1.456	0.015	1.457	1.496	0.039
d(C3,C4)	1.359	1.371	0.012	1.371	1.375	0.004	1.376	1.380	0.004
d(C2,C3)	1.430	1.408	–0.022	1.407	1.399	–0.008	1.398	1.389	–0.009
d(N2,C2)	1.384	1.364	–0.020	1.363	1.357	–0.006	1.357	1.349	–0.008
d(N2,C6)	1.352	1.345	–0.007	1.345	1.345	0.000	1.345	1.348	0.003
d(C5,C6)	1.375	1.383	0.008	1.383	1.386	0.003	1.386	1.390	0.004
d(C4,C5)	1.401	1.389	–0.012	1.389	1.386	–0.003	1.386	1.380	–0.006
V(N1,H1)	1.97	2.08	0.11	-	-	-	-	-	-
V(H1)	-	-	-	0.67	0.71	0.04	-	-	-
V(C1,H1)	-	-	-	-	-	-	1.87	1.97	0.10
V(C1)	0.61	1.28	0.67	1.29	1.15	–0.14	-	-	-
V(C1,C2)	2.86	2.41	–0.45	2.40	2.24	–0.16	2.23	2.09	–0.14
V(C1,P1)	2.31	2.03	–0.28	2.03	1.98	–0.05	1.98	1.94	–0.04
V(N1,Ru)	1.95	1.89	–0.06	3.31	3.65	0.34	3.67	3.77	0.10
V(C1,H2)	2.09	2.07	–0.02	2.07	2.07	0.00	2.07	2.01	–0.06
V(Ru,P1)	2.11	2.12	0.01	2.12	2.15	0.03	2.15	2.14	–0.01
V(Ru,P2)	2.27	2.29	0.02	2.29	2.29	0.00	2.30	2.28	–0.02
V(Ru,C7)	3.22	3.25	0.03	3.24	3.22	–0.02	3.22	3.20	–0.02
V(Ru,H2)	1.74	1.75	0.01	1.75	1.77	0.02	1.77	1.81	0.04
C(Ru)	13.26	13.23	–0.03	13.23	13.22	–0.01	13.22	13.20	–0.02
V(N2,Ru)	3.04	2.83	–0.21	2.82	2.70	–0.12	2.70	2.66	–0.04
V(C3,C4)	3.23	3.06	–0.17	3.06	2.94	–0.12	2.94	2.87	–0.07
V(C2,C3)	2.59	2.77	0.18	2.78	2.91	0.13	2.92	3.02	0.10
V(N2,C2)	2.19	2.30	0.11	2.30	2.37	0.07	2.37	2.42	0.05
V(N2,C6)	2.31	2.38	0.07	2.39	2.43	0.04	2.43	2.46	0.03
V(C5,C6)	3.31	3.16	–0.15	3.16	3.09	–0.07	3.08	3.04	–0.04
V(C4,C5)	2.68	2.83	0.15	2.83	2.87	0.04	2.87	2.88	0.01

Table S23. E_{int}, V_{xc} and V_{cl} (in kcal·mol^{-1}) for selected interactions in the reaction of **1[NH$_2$]** with ammonia.

	E_{int} (kcal·mol^{-1})			V_{xc} (kcal·mol^{-1})			V_{cl} (kcal·mol^{-1})		
	**1-R'**	**1-TS'**	**1-P'**	**1-R'**	**1-TS'**	**1-P'**	**1-R'**	**1-TS'**	**1-P'**
N1–H1	–254.6	–150.8	–15.8	–150.6	–58.9	–4.4	–104.0	–91.9	–11.4
C1–H1	–55.0	–156.0	–161.6	–5.3	–85.6	–169.1	–49.7	–70.4	7.6
Ru–N1	–171.5	–200.8	–212.5	–60.6	–75.1	–86.5	–110.9	–125.7	–126.0
P1–C1	–643.9	–537.1	–435.7	–146.1	–134.8	–124.3	–497.8	–402.3	–311.4
C1–C2	–281.3	–248.4	–216.6	–251.4	–212.9	–195.3	–29.9	–35.5	–21.3
C2–C3	–170.4	–176.9	–181.2	–219.5	–233.5	–240.4	49.2	56.6	59.2
C3–C4	–237.2	–227.6	–223.4	–269.8	–258.5	–253.8	32.6	30.8	30.4
C4–C5	–213.8	–219.9	–222.7	–240.5	–248.8	–252.8	26.7	28.9	30.2
C5–C6	–205.9	–191.9	–184.5	–261.0	–249.4	–242.9	55.1	57.5	58.3
N2–C2	–404.2	–443.2	–448.8	–225.7	–232.3	–236.3	–178.5	–210.9	–212.6
N2–C6	–402.0	–432.3	–440.0	–227.0	–231.9	–235.6	–175.0	–200.4	–204.4
Ru–N2	–215.9	–215.3	–210.1	–77.4	–73.1	–72.0	–138.4	–142.2	–138.1
Ru–P1	29.7	33.2	29.3	–117.1	–115.9	–121.1	146.7	149.1	150.3
Ru–P2	4.9	9.2	10.4	–128.1	–130.4	–131.3	133.1	139.6	141.7
Ru–H2	–145.3	–145.8	–144.3	–131.8	–126.0	–121.2	–13.5	–19.7	–23.1
Ru–C7	–127.7	–122.5	–121.0	–216.2	–216.0	–215.9	88.5	93.5	94.9

Table S24. E_{int}, V_{xc} and V_{cl} (in kcal·mol^{-1}) for selected interactions in the reaction of **1[Cl]** with ammonia.

	E_{int} (kcal·mol^{-1})			V_{xc} (kcal·mol^{-1})			V_{cl} (kcal·mol^{-1})		
	**1-R'**	**1-TS'**	**1-P'**	**1-R'**	**1-TS'**	**1-P'**	**1-R'**	**1-TS'**	**1-P'**
N1–H1	–253.8	–137.9	–24.2	–153.5	–53.3	–6.4	–100.3	–84.6	–17.8
C1–H1	–46.7	–155.9	–162.4	–3.5	–92.5	–165.1	–43.2	–63.4	2.7
Ru–N1	–169.7	–202.5	–213.9	–60.3	–77.0	–87.7	–109.4	–125.6	–126.2
P1–C1	–616.5	–517.1	–437.7	–142.9	–131.7	–124.6	–473.6	–385.3	–313.1
C1–C2	–284.9	–249.1	–217.8	–266.3	–218.9	–197.1	–18.6	–30.2	–20.7
C2–C3	–194.0	–205.2	–211.3	–221.9	–239.7	–247.8	27.9	34.4	36.6
C3–C4	–244.8	–232.7	–228.1	–277.6	–263.3	–258.0	32.7	30.6	29.9
C4–C5	–215.0	–224.2	–227.6	–241.9	–253.0	–257.3	26.9	28.9	29.7
C5–C6	–222.5	–214.5	–212.8	–260.3	–252.0	–249.5	37.8	37.6	36.7
N2–C2	–367.1	–417.1	–433.6	–215.6	–226.5	–233.2	–151.6	–190.6	–200.3
N2–C6	–423.8	–439.5	–431.8	–234.2	–234.7	–234.5	–189.6	–204.8	–197.2
Ru–N2	–210.6	–209.1	–204.7	–76.1	–71.5	–70.8	–134.5	–137.6	–133.9
Ru–P1	26.8	31.4	27.4	–118.3	–116.3	–121.2	145.1	147.7	148.7
Ru–P2	5.3	9.1	10.6	–127.3	–130.1	–130.4	132.7	139.3	141.0
Ru–H2	–144.8	–145.5	–143.8	–132.1	–125.8	–120.9	–12.7	–19.7	–22.9
Ru–C7	–126.4	–120.5	–119.0	–215.4	–215.2	–215.0	89.0	94.7	96.0

Table S25. E_{int}, V_{xc} and V_{cl} (in kcal·mol^{-1}) for selected interactions in the reaction of **1[CH$_3$]** with ammonia.

	E_{int} (kcal·mol^{-1})			V_{xc} (kcal·mol^{-1})			V_{cl} (kcal·mol^{-1})		
	**1-R'**	**1-TS'**	**1-P'**	**1-R'**	**1-TS'**	**1-P'**	**1-R'**	**1-TS'**	**1-P'**
N1–H1	–254.2	–144.3	–21.6	–151.7	–56.1	–6.0	–102.5	–88.2	–15.5
C1–H1	–52.4	–156.7	–162.3	–4.6	–88.9	–166.5	–47.8	–67.8	4.1
Ru–N1	–170.5	–201.6	–212.7	–60.4	–75.9	–86.7	–110.2	–125.7	–126.0
P1–C1	–635.5	–533.8	–441.3	–145.2	–133.8	–125.1	–490.3	–400.0	–316.2
C1–C2	–281.4	–248.3	–216.8	–258.1	–216.4	–196.0	–23.2	–31.9	–20.8
C2–C3	–200.4	–211.8	–218.0	–224.8	–241.1	–249.1	24.4	29.4	31.1
C3–C4	–246.1	–235.7	–231.4	–276.1	–263.5	–258.5	30.0	27.8	27.1
C4–C5	–218.3	–226.8	–230.5	–242.8	–252.9	–257.4	24.5	26.1	27.0
C5–C6	–229.0	–221.7	–219.5	–261.1	–253.6	–250.8	32.1	31.9	31.3
N2–C2	–377.6	–420.7	–434.2	–217.9	–227.0	–233.3	–159.6	–193.7	–200.9
N2–C6	–418.2	–436.1	–431.3	–232.2	–233.6	–234.1	–186.0	–202.4	–197.2
Ru–N2	–215.4	–214.6	–209.5	–77.8	–73.3	–72.0	–137.6	–141.3	–137.5
Ru–P1	28.3	32.9	28.9	–117.8	–116.2	–121.1	146.1	149.1	150.0
Ru–P2	4.9	9.5	10.9	–128.1	–130.4	–130.9	133.1	140.0	141.8
Ru–H2	–145.1	–145.7	–144.3	–131.9	–125.8	–121.0	–13.2	–19.9	–23.2
Ru–C7	–127.4	–121.8	–120.6	–215.6	–215.5	–215.5	88.2	93.7	94.9

Table S26. E_{int}, V_{xc} and V_{cl} (in kcal·mol^{-1}) for selected interactions in the reaction of **1[OH]** with ammonia.

	E_{int} (kcal·mol^{-1})			V_{xc} (kcal·mol^{-1})			V_{cl} (kcal·mol^{-1})		
	**1-R'**	**1-TS'**	**1-P'**	**1-R'**	**1-TS'**	**1-P'**	**1-R'**	**1-TS'**	**1-P'**
N1–H1	–254.2	–148.1	–18.7	–151.9	–57.9	–4.9	–102.3	–90.1	–13.9
C1–H1	–51.2	–154.8	–161.9	–4.4	–86.5	–167.8	–46.7	–68.3	5.9
Ru–N1	–170.6	–201.1	–212.6	–60.3	–75.4	–87.0	–110.3	–125.7	–125.6
P1–C1	–631.8	–534.4	–435.7	–145.3	–134.6	–125.2	–486.4	–399.8	–310.5
C1–C2	–283.0	–249.6	–216.0	–258.0	–215.9	–195.7	–25.0	–33.7	–20.2
C2–C3	–159.4	–164.8	–169.4	–218.6	–234.5	–242.8	59.2	69.7	73.4
C3–C4	–238.6	–227.3	–222.3	–273.6	–260.1	–254.6	35.0	32.8	32.2
C4–C5	–210.4	–218.3	–221.6	–237.9	–248.9	–253.7	27.5	30.6	32.1
C5–C6	–195.5	–178.8	–172.5	–264.0	–250.7	–245.0	68.5	71.9	72.5
N2–C2	–393.6	–437.4	–445.8	–222.9	–230.2	–235.0	–170.7	–207.1	–210.8
N2–C6	–404.1	–435.4	–439.3	–227.5	–232.4	–235.0	–176.6	–203.0	–204.3
Ru–N2	–215.7	–214.2	–209.5	–77.9	–73.0	–72.4	–137.8	–141.2	–137.1
Ru–P1	28.5	32.9	28.2	–117.1	–115.5	–120.7	145.6	148.4	148.9
Ru–P2	5.2	9.3	10.6	–127.2	–129.7	–130.2	132.4	138.9	140.9
Ru–H2	–145.2	–145.8	–144.1	–132.0	–126.1	–121.1	–13.2	–19.7	–23.0
Ru–C7	–127.6	–122.1	–120.5	–216.0	–215.9	–215.5	88.4	93.8	95.0

Table S27. E_{int}, V_{xc} and V_{cl} (in kcal·mol^{-1}) for selected interactions in the reaction of **1[COCH$_3$]** with ammonia.

	E_{int} (kcal·mol^{-1})			V_{xc} (kcal·mol^{-1})			V_{cl} (kcal·mol^{-1})		
	**1-R'**	**1-TS'**	**1-P'**	**1-R'**	**1-TS'**	**1-P'**	**1-R'**	**1-TS'**	**1-P'**
N1–H1	–256.0	–140.2	–22.7	–151.6	–54.0	–6.1	–104.3	–86.2	–16.5
C1–H1	–43.6	–156.4	–162.3	–2.1	–91.5	–166.2	–41.5	–64.9	3.8
Ru–N1	–170.6	–202.3	–212.8	–60.1	–76.6	–86.7	–110.5	–125.7	–126.0
P1–C1	–616.6	–518.9	–434.7	–143.0	–132.2	–124.4	–473.6	–386.6	–310.2
C1–C2	–285.5	–248.3	–216.7	–266.2	–217.3	–196.4	–19.2	–31.0	–20.4
C2–C3	–166.9	–173.4	–177.5	–221.0	–240.1	–248.0	54.1	66.7	70.5
C3–C4	–236.8	–223.2	–217.0	–276.5	–261.2	–254.9	39.6	38.0	37.9
C4–C5	–208.1	–215.9	–218.4	–240.5	–252.1	–256.5	32.5	36.2	38.1
C5–C6	–192.6	–180.2	–177.4	–260.6	–251.0	–248.2	68.0	70.8	70.8
N2–C2	–374.0	–421.7	–435.9	–216.2	–226.9	–233.3	–157.9	–194.8	–202.6
N2–C6	–424.5	–442.1	–437.3	–233.5	–234.6	–234.7	–191.0	–207.5	–202.6
Ru–N2	–215.2	–212.4	–208.7	–77.6	–73.2	–72.6	–137.5	–139.3	–136.1
Ru–P1	28.3	31.9	28.6	–118.0	–116.0	–120.5	146.2	147.8	149.1
Ru–P2	7.3	9.0	10.5	–125.8	–130.2	–130.6	133.1	139.2	141.0
Ru–H2	–145.3	–145.4	–143.9	–131.9	–125.9	–121.4	–13.4	–19.4	–22.5
Ru–C7	–126.6	–121.0	–119.3	–215.6	–215.0	–214.6	89.0	94.1	95.4

Table S28. QTAIM charges (in |e$^-$|) for relevant atoms in the reaction of **1[NH$_2$]** with ammonia.

	**1-R'**	**1-TS'**	**1-P'**
H1	0.40	0.35	0.08
N1	–1.03	–1.11	–1.12
C1	–0.89	–0.77	–0.52
Ru	0.67	0.70	0.70
P1	1.82	1.75	1.74
C2	0.43	0.48	0.50
C3	0.36	0.38	0.39
C4	–0.04	–0.03	–0.03
C5	0.33	0.36	0.38
C6	0.43	0.46	0.48
N2	–1.15	–1.16	–1.14

Table S29. QTAIM charges (in |e⁻|) for relevant atoms in the reaction of **1[Cl]** with ammonia.

	**1-R’**	**1-TS’**	**1-P’**
H1	0.39	0.34	0.12
N1	–1.02	–1.11	–1.12
C1	–0.83	–0.73	–0.52
Ru	0.67	0.69	0.70
P1	1.82	1.74	1.73
C2	0.40	0.47	0.51
C3	0.06	0.08	0.08
C4	0.04	0.04	0.05
C5	0.06	0.07	0.08
C6	0.49	0.51	0.50
N2	–1.13	–1.13	–1.11

Table S30. QTAIM charges (in |e⁻|) for relevant atoms in the reaction of **1[CH$_3$]** with ammonia.

	**1-R’**	**1-TS’**	**1-P’**
H1	0.40	0.34	0.10
N1	–1.02	–1.11	–1.12
C1	–0.88	–0.76	–0.53
Ru	0.67	0.70	0.70
P1	1.82	1.75	1.73
C2	0.39	0.44	0.47
C3	–0.02	–0.01	0.00
C4	–0.04	–0.03	-0.03
C5	–0.02	–0.01	0.00
C6	0.44	0.46	0.46
N2	–1.15	–1.16	–1.13

Table S31. QTAIM charges (in |e⁻|) for relevant atoms in the reaction of **1[OH]** with ammonia.

	**1-R’**	**1-TS’**	**1-P’**
H1	0.40	0.35	0.10
N1	–1.02	–1.11	–1.12
C1	–0.86	–0.76	–0.51
Ru	0.67	0.70	0.70
P1	1.82	1.75	1.73
C2	0.43	0.49	0.51
C3	0.49	0.51	0.52
C4	–0.04	–0.03	–0.03
C5	0.47	0.50	0.52
C6	0.44	0.48	0.50
N2	–1.14	–1.16	–1.13

Table S32. QTAIM charges (in |e⁻|) for relevant atoms in the reaction of **1[COCH$_3$]** with ammonia.

	**1-R’**	**1-TS’**	**1-P’**
H1	0.41	0.34	0.11
N1	–1.02	–1.11	–1.12
C1	–0.82	–0.73	–0.51
Ru	0.67	0.69	0.70
P1	1.82	1.74	1.73
C2	0.41	0.48	0.50
C3	0.44	0.47	0.48
C4	0.02	0.03	0.04
C5	0.43	0.47	0.48
C6	0.49	0.51	0.51
N2	–1.13	–1.14	–1.12

Comparison between tert-butyl and methyl-substituted phosphine ligands

Table S33. Reaction and activation electronic and Gibbs energies (relative to **1-^{t}Bu-R’**) for the reaction of the tert-butyl-substituted system with the complete set of amines.

	ΔE (kcal·mol^{-1})				ΔG (kcal·mol^{-1})			
Amine	**1-^{t}Bu**	**1-^{t}Bu -R’**	**1-^{t}Bu -TS’**	**1-^{t}Bu -P’**	**1-^{t}Bu**	**1-^{t}Bu -R’**	**1-^{t}Bu -TS’**	**1-^{t}Bu -P’**
**NH$_3$**	12.9	0.0	**23.1**	**17.2**	–3.7	0.0	**20.0**	**16.1**
**^{i}Pr-NH$_2$**	11.7	0.0	**27.0**	**21.6**	–6.9	0.0	**23.6**	**20.8**
**2,4-(CF$_3$)$_2$-Ph-NH$_2$**	6.1	0.0	**8.7**	–**14.6**	–11.6	0.0	**6.6**	–**14.2**
**A**	11.9	0.0	**24.3**	**19.2**	–5.2	0.0	**21.6**	**18.6**
**B**	14.4	0.0	**26.1**	**19.8**	–2.8	0.0	**23.6**	**19.0**
**C**	14.8	0.0	**26.1**	**20.6**	–2.5	0.0	**23.6**	**19.9**
**D**	13.2	0.0	**25.6**	**18.2**	–4.3	0.0	**21.6**	**17.4**
**E**	12.4	0.0	**24.9**	**17.0**	–4.6	0.0	**22.3**	**16.4**
**F**	13.8	0.0	**24.3**	**16.9**	–4.1	0.0	**20.0**	**16.1**
**G**	16.8	0.0	**25.1**	**15.6**	–1.2	0.0	**22.1**	**14.8**
**H**	10.4	0.0	**19.6**	**12.0**	–6.5	0.0	**17.5**	**11.9**
**I**	8.7	0.0	**14.8**	–**0.6**	–8.1	0.0	**13.0**	–**1.0**
**J**	9.7	0.0	**17.0**	**0.3**	–8.2	0.0	**13.9**	–**0.4**
**K**	8.7	0.0	**14.8**	–**12.8**	–9.5	0.0	**12.1**	–**12.0**
**L**	10.5	0.0	**15.0**	–**6.1**	–6.4	0.0	**13.8**	–**4.3**
**M**	7.3	0.0	**12.7**	**-10.5**	–10.1	0.0	**10.4**	–**10.0**
**N**	8.6	0.0	**14.7**	–**5.2**	–9.3	0.0	**12.1**	–**5.3**
**O**	10.3	0.0	**14.2**	–**17.5**	–6.0	0.0	**13.4**	–**15.2**

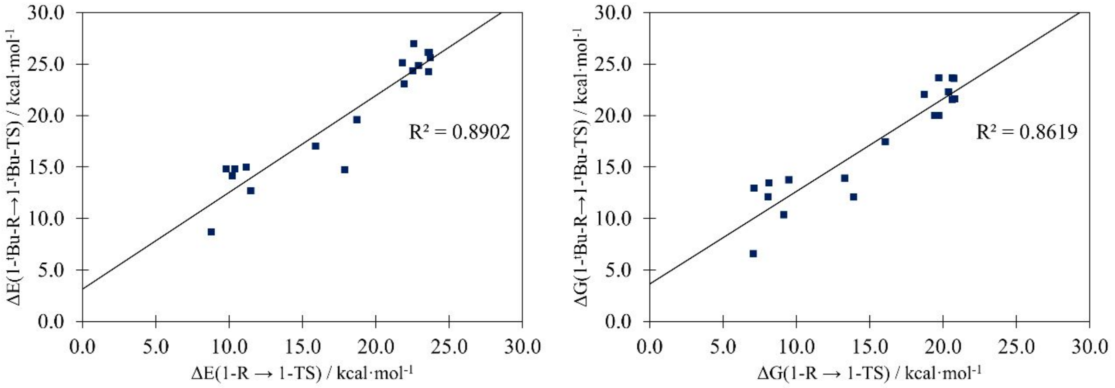

Figure S2. Activation energies (left) and Gibbs energies (right) obtained for the tert-butyl-substituted system (*y* axis) versus those obtained for the methyl-substituted one (*x* axis).

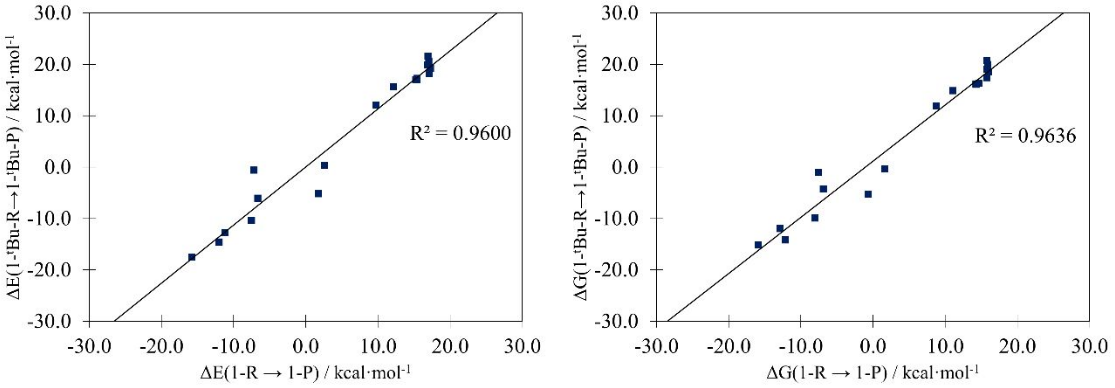

Figure S3. Reaction energies (left) and Gibbs energies (right) obtained for the tert-butyl-substituted system (*y* axis) versus those obtained with for methyl-substituted one (*x* axis).
